# Molecular vasculogenic mimicry–Related signatures predict clinical outcomes and therapeutic responses in bladder cancer: Results from real-world cohorts

**DOI:** 10.3389/fphar.2023.1163115

**Published:** 2023-04-24

**Authors:** Chunyu Zhang, Jiatong Xiao, Tong Yuan, Yunbo He, Dingshan Deng, Zicheng Xiao, Jinbo Chen, Xiongbing Zu, Peihua Liu, Zhi Liu

**Affiliations:** ^1^ Departments of Urology, Xiangya Hospital, Central South University, Changsha, China; ^2^ National Clinical Research Center for Geriatric Disorders, Xiangya Hospital, Changsha, Hunan, China; ^3^ Hepatic Surgery Center, Tongji Hospital, Tongji Medical College, Huazhong University of Science and Technology, Wuhan, China; ^4^ Department of Urology, The Second Affiliated Hospital, Guizhou Medical University, Kaili, China

**Keywords:** bladder cancer, vasculogenic mimicry, molecular subtype, tumor microenvironment, immunotherapy

## Abstract

Bladder cancer (BLCA) is a heterogeneous disease, and there are many classical molecular subtypes that reflect tumor immune microenvironment (TME) heterogeneity but their clinical utility is limited and correct individual treatment and prognosis cannot be predicted based on them. To find reliable and effective biomarkers and tools for predicting patients’ clinical responses to several therapies, we developed a new systemic indicator of molecular vasculogenic mimicry (VM)–related genes mediated by molecular subtypes based on the Xiangya cohort and additional external BLCA cohorts using a random forest algorithm. A correlation was then done between the VM_Score and classical molecular subtypes, clinical outcomes, immunophenotypes, and treatment options for BLCA. With the VM_Score, it is possible to predict classical molecular subtypes, immunophenotypes, prognosis, and therapeutic potential of BLCA with high accuracy. The VM_Scores of high levels indicate a more anticancer immune response but a worse prognosis due to a more basal and inflammatory phenotype. The VM_Score was also found associated with low sensitivity to antiangiogenic and targeted therapies targeting the FGFR3, β-catenin, and PPAR-γ pathways but with high sensitivity to cancer immunotherapy, neoadjuvant chemotherapy, and radiotherapy. A number of aspects of BLCA biology were reflected in the VM_Score, providing new insights into precision medicine. Additionally, the VM_Score may be used as an indicator of pan-cancer immunotherapy response and prognosis.

## 1 Introduction

Bladder cancer is one of the most common urinary malignancies causing an estimated 81,180 new cases and 17,100 deaths in 2022 (Siegel et al., 2022). Surgery, chemotherapy, and targeted therapy are the effective treatments for BLCA, but most patients have a poor prognosis (Witjes et al., 2021). Due to the high immunogenicity of BLCA, the application of tumor immunotherapy, especially immune checkpoint inhibitors (ICI), has achieved certain results, but the overall efficacy is still not ideal (Witjes et al., 2021). This is because there are no reliable or effective biomarkers or tools for predicting patients’ clinical responses to these therapies, and only a minority of patients with BLCA respond to these therapies. Therefore, it is crucial to develop biomarkers for precise diagnosis and treatment.

There are three components to the tumor immune microenvironment (TME): cancer cells, immune cells, and the extracellular matrix. There can be differences in clinical responses to treatment based on the level of TME heterogeneity in patients with the same pathological stage and grade (Dagogo-Jack and Shaw, 2018). It is worth noting that high TME heterogeneity hinders the realization of BLCA precision medicine. As a result, understanding TME heterogeneity can shed light on many aspects of bladder cancer biology and lead to more effective bladder cancer treatment. The TME could be a promising pathway to precision medicine in BLCA through the development of new therapeutic response prediction markers and therapeutic target heterogeneity.

Vasculogenic mimicry (VM) has recently been detected in a number of malignant tumors that provide a novel strategy for treating them clinically. VM vessels are composed of endothelial tumor cells, periodic acid–Schiff (PAS)–positive cells, and rich external matrix components (Maniotis et al., 1999). Nutrients and oxygen-carrying red blood cells are delivered to tumors by VM. A number of mechanisms have been proposed for VM formation, which include cancer stem cells (CSCs) and epithelial–mesenchymal transformation (EMT), as well as various signaling pathways that promote VM formation, such as matrix metalloproteinases (MMPs), focal adhesion kinase (FAK), vascular endothelial (VE)–cadherin, phosphatidylinositol 3-kinase (PI3K), and hypoxia inducible factor 1a (HIF-1a) (Lu et al., 2013; Sun et al., 2017). In addition to liver cancer, ovarian cancer, gastric cancer, prostate cancer, and nasopharyngeal cancer, VM has been observed in many other cancers as well. According to a large number of clinical studies, VM is strongly associated with tumor invasiveness and poor prognosis (Seftor et al., 2012; Zhang et al., 2019). However, the significance of VM for the diagnosis, molecular typing, and treatment of BLCA remains unclear.

VM is associated with tumor microenvironments such as CSC, cancer fibroblasts, tumor-associated macrophages, and hypoxia (Luo et al., 2020). To date, the role of molecular VM-related genes in shaping TME heterogeneity remains unclear in BLCA. A correlation was assessed between the mRNA levels of these VM-related molecules and the heterogeneity of the TME, immunophenotype, clinical characteristics of the disease, and response to BLCA treatment. In order to quantify these subtypes in BLCA, the VM_Score was generated using a molecular subtype system mediated by molecular VM-related genes.

## 2 Material and methods

### 2.1 Workflow

**FIGURE 1:**
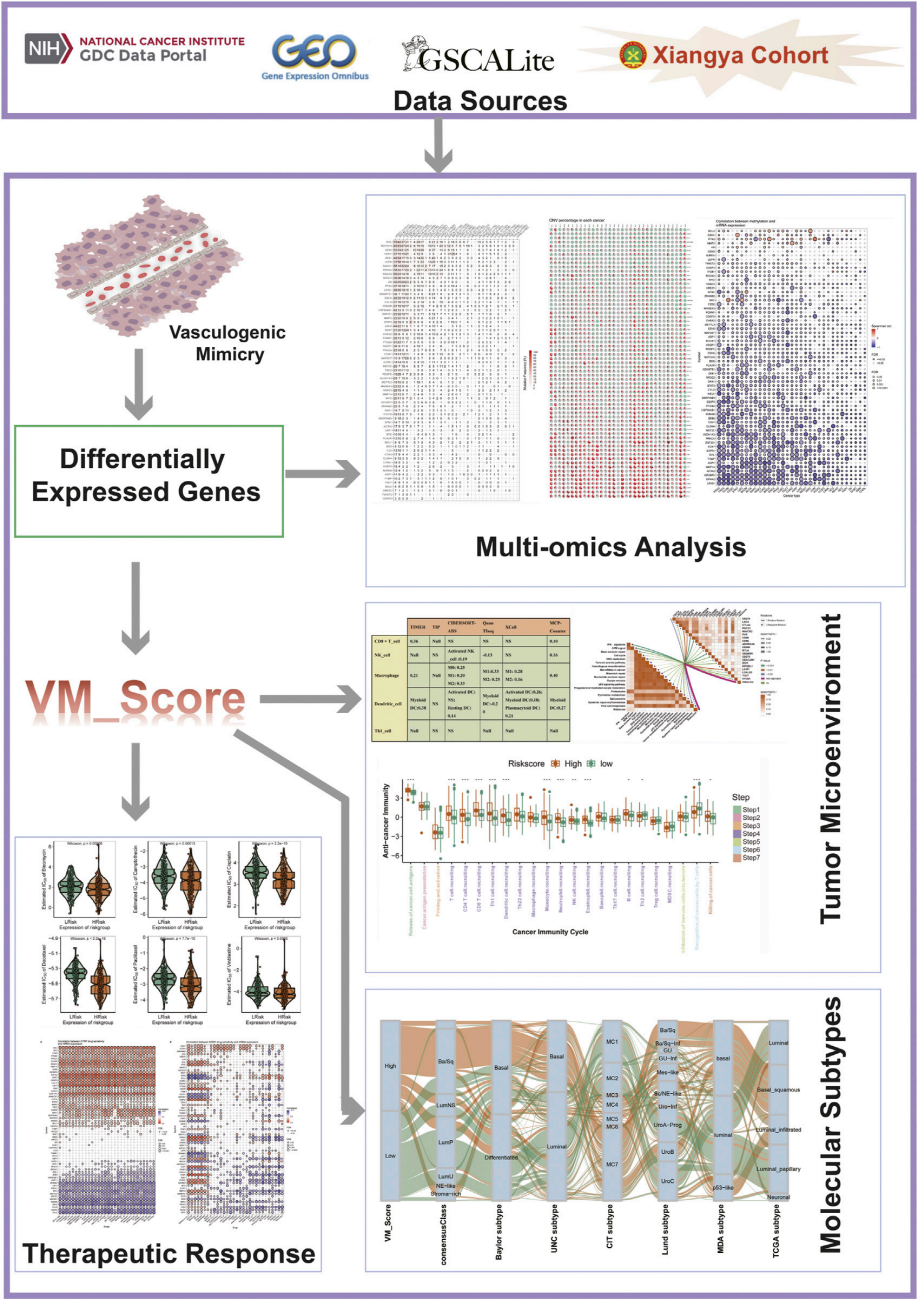
Flow chart.

The workflow of our research is shown in Figure 1.

### 2.2 Data retrieval and preprocessing

#### 2.2.1 External public cohorts

A cohort from the Cancer Genome Atlas (TCGA) was downloaded for RNA sequencing data (FPKM values) and clinical data (https://portal.gdc.cancer.gov/), and FPKM was subsequently converted to TPM value. A total of 412 BLCA samples were included in this study after genomic and clinicopathological data were filtered.

The GSE32894 dataset was downloaded from Gene Expression Omnibus (GEO), and 224 BLCA sample were included in this study. The GSE13507 and GSE48075 datasets were downloaded from GEO, 165 and 73 BLCA sample were included in this study respectively.

#### 2.2.2 Xiangya cohort

Preprocessing and data analysis have been well described in our previous studies (Liu et al., 2021).

### 2.3 Determination of molecular VM-related gene sets

There is no comprehensive overview of molecular VM-related genes at present. The following search term was used in PubMed: vasculogenic mimicry to retrieve all genes associated with molecular VM. Ultimately, a total of 182 molecular VM-related genes were collected (Supplementary Table S1).

The identification of differentially expressed molecular VM-related genes (molecular VM related DEGs) and functional analysis.

To identify molecular VM-related DEGs in BLCA tissue when compared to normal tissue, the empirical Bayesian method of the limma R package was used (Supplementary Table S2). For molecular VM-related DEGs, the screening criteria were |log (fold change) |>1 and adjusted *p*-value <0.05. Based on the aforementioned molecular VM-related DEGs, the Kyoto Encyclopedia of Genes and Genomes (KEGG) and Gene Ontology (GO) analyses were performed (Yu et al., 2012).

### 2.4 Development and validation of VM_Score

We used the “rfsrc” function from the “randomForestSRC” package to develop a risk score based on molecular VM-related DEGs (Supplementary Table S3). The median value of the VM_Score was used as the cutoff value, and patients were divided into high- and low-risk groups based on their VM_Scores. A survival curve was plotted using the Kaplan–Meier (K-M) method; the log–rank test was implemented in the “survminer” R package; and a predictive accuracy assessment of the risk score was conducted using the timeROC function provided in the “tROC” R package. Additionally, univariate and multivariate Cox analyses were conducted on the TCGA-BLCA cohort to analyze the independent effects of gender, age, stage, and VM_Score on prognosis. An independent prognostic predictive value nomogram was plotted based on these factors. ROC and calibration curves were used to validate the nomogram.

### 2.5 Description of immunological features in BLCA TME

The anticancer immune response in the BLCA TME consisted of several steps: cancer antigen release and presentation (Steps 1 and 2), anticancer immune priming and activation (Step 3), immune cell trafficking (Step 4), immune cell infiltration into the TME (Step 5), T cell recognition of cancers (Step 6), and killing of cancer cells (Step 7) (Chen and Mellman, 2013). The seven steps determined the fate of tumor cells. In addition, we employed several independent algorithms to calculate the levels of tumor-infiltrating immune cells (TIICs) based on RNA-seq data, which included TIMER, TIP, quanTIseq, xCell, CIBERSORT-ABS, and MCP-counter (Newman et al., 2015; Becht et al., 2016; Li et al., 2016; Xu et al., 2018; Finotello et al., 2019; Li et al., 2020). The T-cell inflammation score (TIS), which predicts clinical response to immune checkpoint blockade (ICB), can reflect pre-existing anticancer immunity in TMEs (Ayers et al., 2017). In this study, we calculated the enrichment scores for several immunotherapy response-related pathways using ssGSEA. A total of 20 inhibitory immune checkpoints were screened and collected, which included PD-1, TIGIT, LAG3-PD-L1, and CTLA-4. In our previous research, these immunological characteristics have been well described (Hu et al., 2021a; Hu et al., 2021b).

### 2.6 Prediction of molecular subtypes in BLCA

A highly heterogeneous tumor, BLCA can be treated with precision medicine when individual molecular subtypes can be identified. Previous studies have clarified molecular subtype systems as a result, such as CIT, Lund, TCGA, Baylor, MDA, UNC, and consensus subtypes (Sjödahl et al., 2012; Choi et al., 2014; Damrauer et al., 2014; Rebouissou et al., 2014; Robertson et al., 2017; Mo et al., 2018; Kamoun et al., 2020). As a starting point for subtyping molecular structures, we used the packages ConsensusMIBC and BLCAsubtyping in the R language. A total of 12 BLCA heterogeneous signatures were also collected. These BLCA-specific features and molecular subtypes were correlated further with the VM_Score. As a result of the initial assignment of all samples to basal or luminal subtypes, the ROC curves were plotted to determine the accuracy of the VM_Score in predicting the molecular subtypes.

### 2.7 Gene set variation analysis

As an unsupervised and non-parametric method, the gene set variation analysis (GSVA) can be used to estimate activity differences of pathways or biological processes in expression of data set samples. The differences of 50 correlation pathways in the VM_Score were investigated using the “GSVA” R package (Hänzelmann et al., 2013).

### 2.8 Response prediction of BLCA therapy

Because chemotherapy is critical for patients with end-stage BLCA, the pRRophetic software package was used to assess the efficacy of six commonly used chemotherapeutics (cisplatin, bleomycin, camptothecin, docetaxel, paclitaxel, and vinblastine). High-risk and low-risk score groups were compared in terms of 50% inhibitory concentration (IC50) of the six chemotherapeutic agents listed above. Also, GSCALite was used to analyze VM-related DEGs and chemotherapeutic drug sensitivity (http://bioinfo.life.hust.edu.cn/web/GSCALite/) (Liu et al., 2018). It is also important to consider targeted therapy, radiation therapy, and alternative treatment options. This led to the collection of several potential predictors of response to radiation therapy and targeted therapy.

### 2.9 Statistical analysis

In order to visualize data and perform statistical analysis, the R software (version 4.0.5) was used. In order to analyze the relationship between the continuous variables, we used Pearson’s or Spearman’s correlations. We used the t-test when the continuous variables fit the normal distribution. Otherwise, we use the Mann–Whitney U test. To assess risk score association with prognosis, univariate and multivariate Cox regression analyses were performed. With the help of the ROC curves, the accuracy of the VM_Score was calculated in terms of prognosis and molecular subtype prediction. We used *p* < 0.05 for all statistical tests and two-sided tests for all analyses.

## 3 Results

### 3.1 Landscape and functional analysis of VM-related DEGs in BLCA

Between BLCA and normal tissue, 66 DEGs associated with VM were screened. Among them, 28 molecular VM-related genes were highly expressed in BLCA, and 38 molecular VM-related genes had low expression (Supplementary Figures S1A, B). The GO and KEGG enrichment analysis showed that molecular VM-related DEGs were enriched in several pathways, such as, cell junction assembly, tissue remodeling, regulation of anoikis, regulation of peptidase activity, and focal adhesion. Notably, molecular VM-related DEGs were mainly associated with cell junction, adhesion, and anoikis (Supplementary Figures S1C, D). A novel focal adhesion–related gene signature was significantly correlated with tumor grade, tumor stage, immune scores, and immune infiltrate types, according to Lin et al. (2021). Anoikis-resistant mechanically stressed cancer cells exhibited enhanced motility and evaded immune surveillance by natural killer cells, according to Fanfone et al. (2022). This PPI network shows that these molecular VM-related DEGs are closely related to each other as shown in Supplementary Figure S1E.

### 3.2 Pan-cancer multiomics analysis of VM DEGs

In addition, we analyzed 66 molecular VM-related DEGs in pan-cancer based on multi-omics signatures. It was observed that these genes were significantly mutated in the following cancers: UCEC, SKCM, COAD, STAD, DLBC, READ, and BLCA. Among them, the SVIL gene had the highest mutation frequency in UCEC, at 76% (Supplementary Figure S2). Heterozygous amplifications and deletions are the main types of copy number variations in molecular VM-related DEGs in pan-cancer, among which the copy numbers of NTN1, SIPR1, LAMTOR5, and ZRANB2 are heterozygous deletions in most tumors. By contrast, EZH2, CDK5, AQP1, and AURKA are heterozygously amplified in most tumors (Supplementary Figure S3). It has been found that the gene copy number variation is one of the most important factors affecting the expression of molecular VM molecules. Most tumor types show a positive correlation between CNV and mRNA expression. There is a significant positive correlation between CNV and PTK2 mRNA expressions (Supplementary Figure S4). As a result, the methylation levels of molecular VM genes are negatively correlated with the levels of mRNA expression in most cancers (Supplementary Figure S5).

### 3.3 Development and validation of VM_Score in BLCA cohorts

**FIGURE 2:**
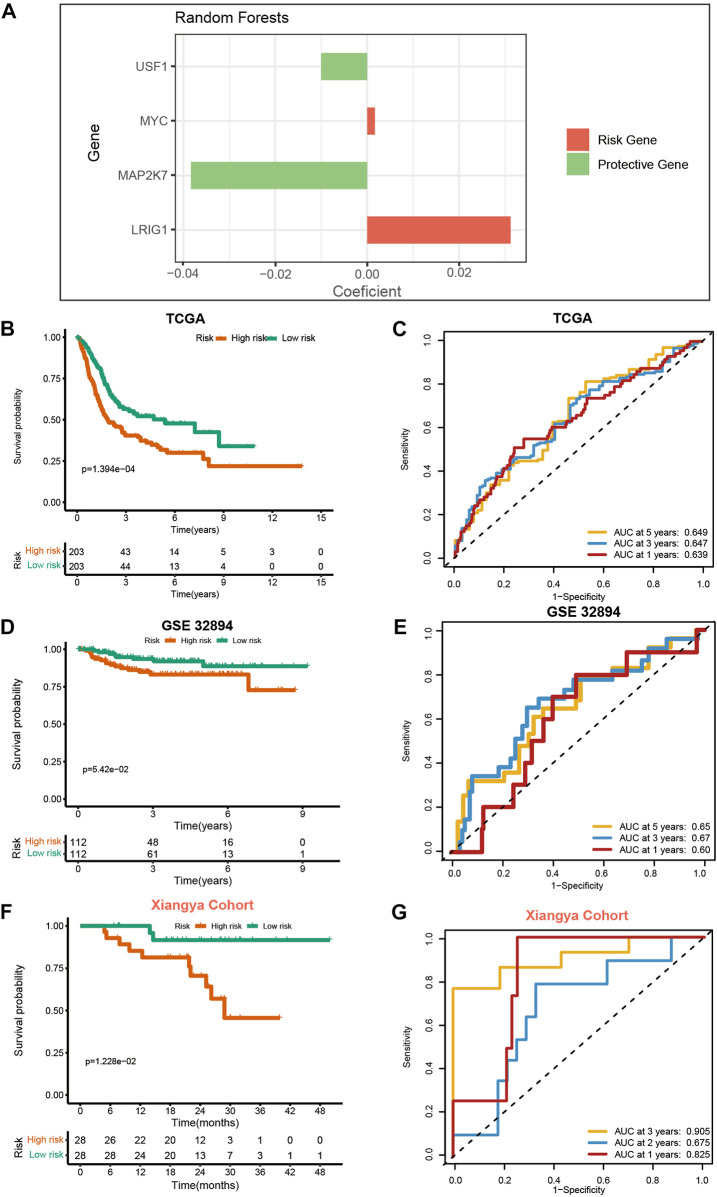
Development and validation of VM_Score in multiple BLCA cohorts. **(A)** Four best candidates were screened by random forest algorithm to determine the generation of VM_Score. **(B)** Kaplan–Meier analysis of OS for VM_Score in TCGA-BLCA cohort. **(C)** ROC curves of VM_Score for predicting OS in TCGA-BLCA cohort. **(D,E)** Validation of VM_Score in GSE32894. **(F,G)** Validation of VM_Score in Xiangya cohort.

We generated the VM_Score in the TCGA cohort by using the random forest algorithm (Figure 2A). Meanwhile, mRNA expression levels of these four target genes were verified in cell lines and specimens (Supplementary Figure S16). The BLCA cohort from the TCGA was divided into two risk groups based on their level of risk. Notably, we successfully validated that BLCA patients had a worse prognosis in the high-risk group (Figure 2B). More importantly, the VM_Score predicted 1-, 3-, and 5-year OS with accuracies of 0.639, 0.647, and 0.649, respectively (Figure 2C).

To assess the extrapolation of risk scores, we validated the risk scores using the external cohort GSE32894, GSE13507, and GSE48075 (Figures 2D, E, and Supplementary Figure S17). In the GSE32894 cohort, patients with a higher VM_Score also had poorer survival outcomes, with prediction accuracies of 0.60, 0.67, and 0.65 at 12, 36, and 60 months, respectively. In addition to this, we constructed our own Xiangya cohort by sequencing and found that the results are consistent with the above trends and have better predictive functions. At 12, 24, and 36 months, patients with high-risk scores had poorer survival outcomes (*p* = 1.228e−02), with a prediction accuracy of 0.825, 0.675, and 0.905, respectively (Figures 2F, G). All these results suggest that the prediction accuracy of the VM_Score is relatively high and may be a powerful predictive tool for the BLCA operating system.

### 3.4 Relationship between VM_score and clinicopathological features

**FIGURE 3:**
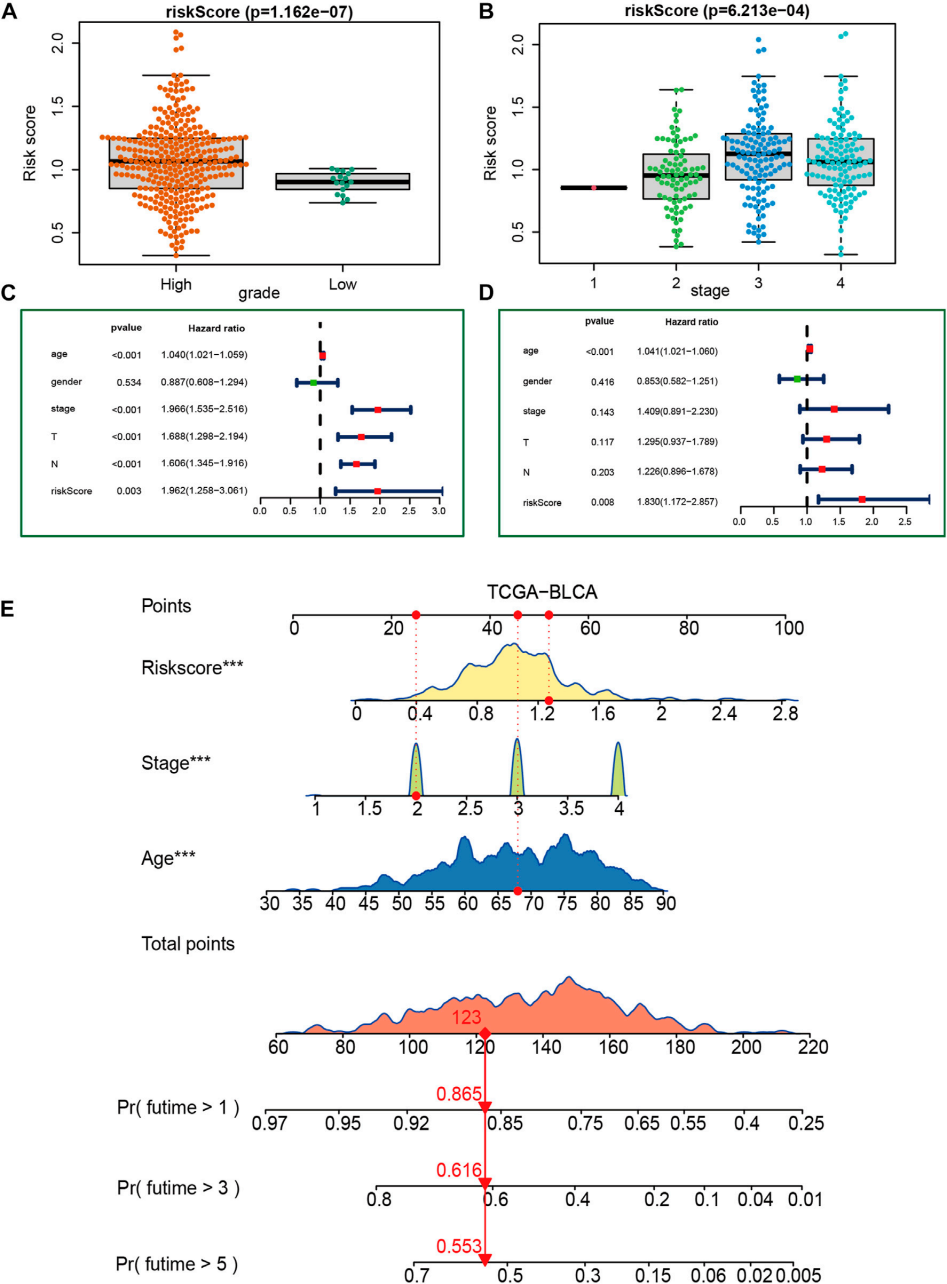
Construction of a nomogram in the TCGA-BLCA cohort. **(A, B)** Relationship between VM_Score and tumor grade and stage in TCGA-BLCA cohort. **(C, D)** Results of univariate and multivariate Cox analyses. **(E)** Nomogram developed based on stage, age, and VM_Score to predict overall survival at 1, 3, and 6 years.

**FIGURE 4:**
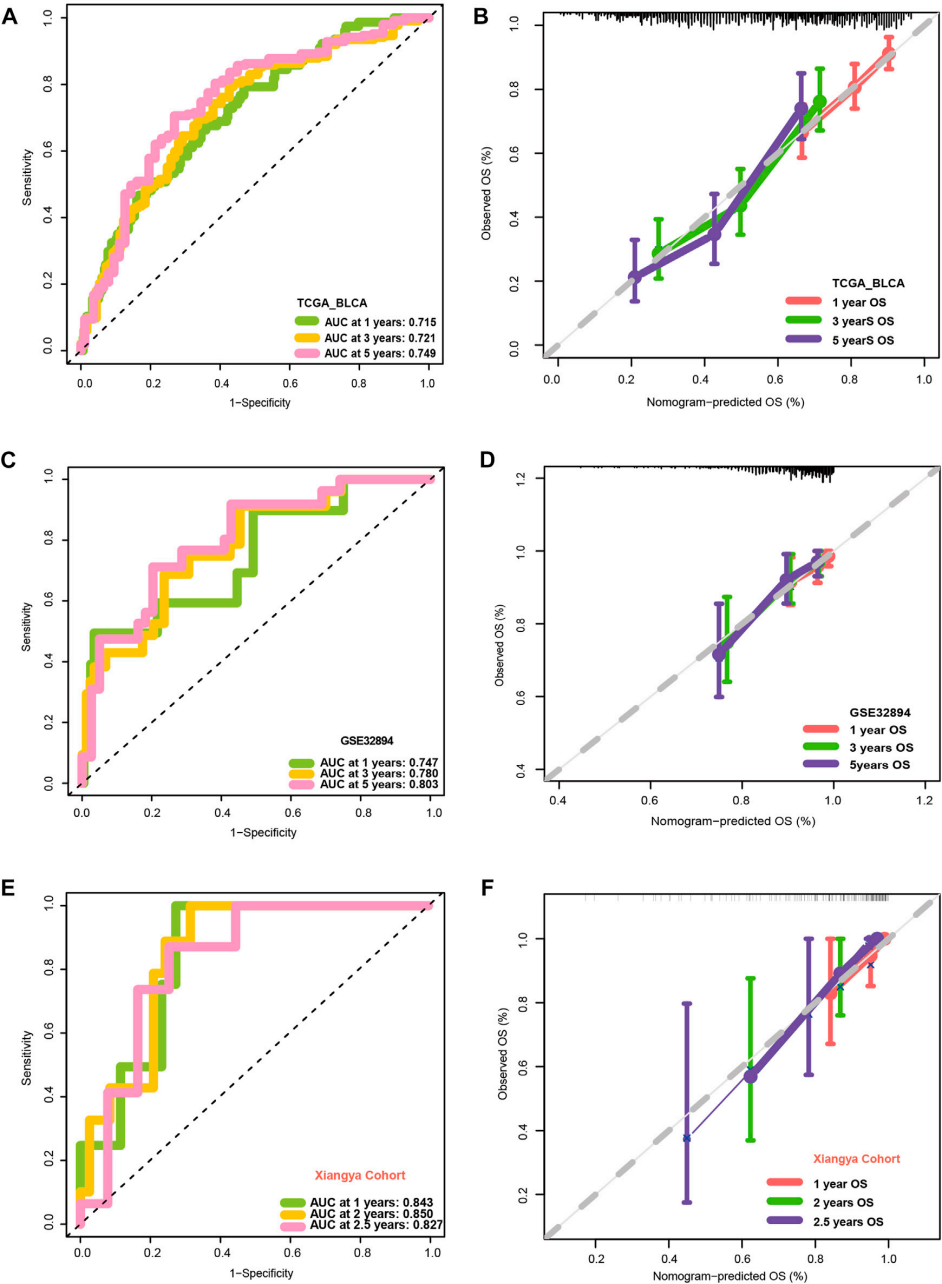
Validation of multiple cohorts of VM_Score. **(A)** ROC curves of the nomogram. **(B)** Calibration curves of the nomogram measured using the Hosmer–Lemeshow test. **(C, D)** Validity of VM_Score in GSE32894. **(E, F)** Validity of VM_Score in Xiangya cohort.

Figures 3A, B show that patients with higher grades and stages have higher risk scores, which is consistent with the prognostic correlation of the VM_Score. A subgroup analysis was performed by stage, sex, and age, and as expected, high-risk patients had a worse prognosis in virtually all subgroups (Supplementary Figure S6). The univariate Cox analysis also revealed that the VM_Score, stage, and age predict prognosis significantly (Figure 3C). It was also confirmed by multivariate Cox analysis that the VM_Scores were independent prognostic factors (Figure 3D). As a result, stage no longer serves as a reliable prognostic predictor in the case of patients with BLCA. The results in this study confirm that VM_Score is a reliable indicator of prognosis for BLCA patients. The purpose of this study was to improve the predictive value of the VM_Score for BLCA prognosis, we combined the VM_Score with age, tumor stage, and other factors with prognostic value in the univariate Cox regression analysis to establish a nomogram (Figure 3E). We further validated the accuracy of the nomogram in predicting BLCA prognosis using the ROC and calibration curves. The nomogram’s predictive accuracy for 1-, 3-, and 5-year OS in the TCGA-BLCA cohort was 0.715, 0.721, and 0.749, respectively (Figure 4A). This accuracy of the nomogram and its clinical significance are illustrated by the calibration curve showing that the OS predicted by the nomogram is highly consistent with the actual OS (Figure 4B). Furthermore, both validation sets of the GSE32894 and Xiangya cohort showed high accuracy of the nomograms (Figures 4C–F).

### 3.5 Prediction of VM_score for immune phenotypes and clinical response of ICB in BLCA

**FIGURE 5:**
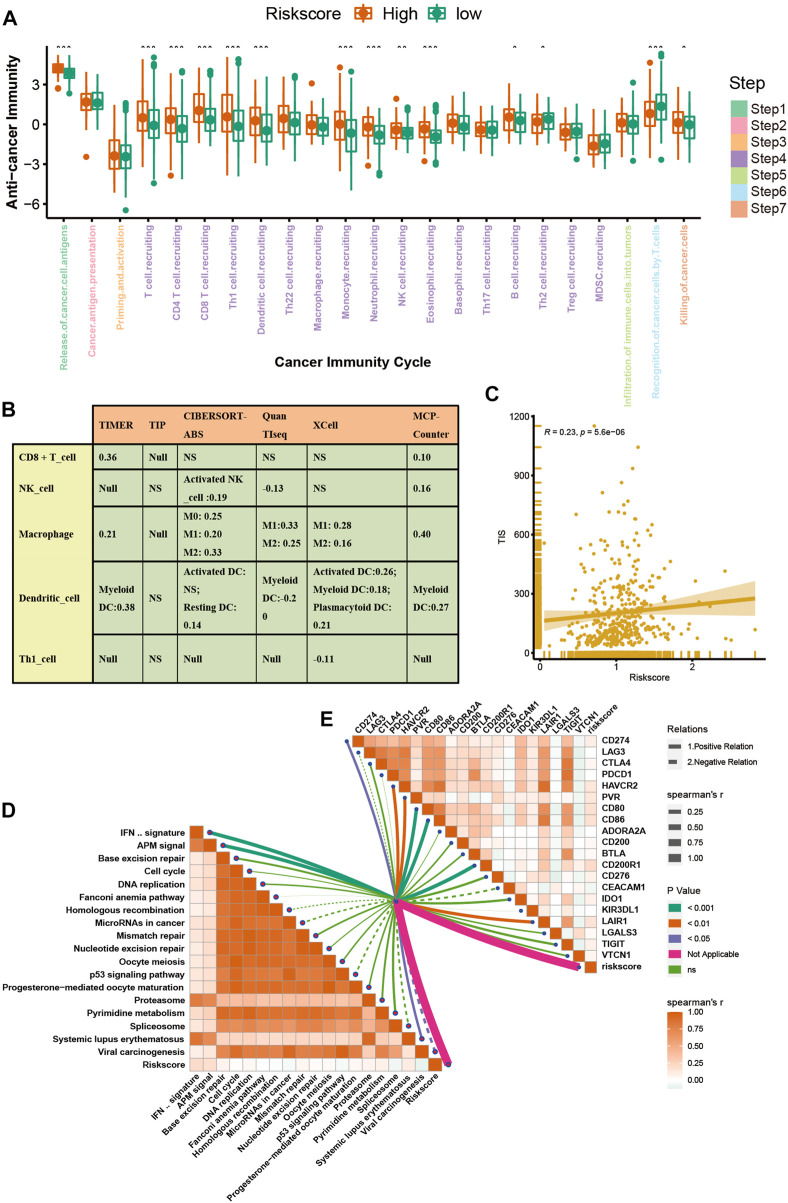
VM_Score correlated with the tumor immune microenvironment characteristics. **(A)** Differences in cancer immune cycling activity between high- and low-risk groups. **(B)** Relationship between VM_Score and several immune cells (CD8^+^T cells, NK cells, macrophages, Th1 cells, and DCs) in six independent algorithms. **(C)** Relationship between VM_Score and T cell inflamed score (TIS). **(D)** Correlation between VM_Score and enrichment of ICB response–related pathways. **(E)** Relationship between VM_Score and immune checkpoints (*p < 0.05; **p < 0.01; ***p < 0.001).

The advent of immunotherapy has brought light to patients with advanced bladder cancer, but the efficacy of immunotherapy or neoadjuvant therapy largely depends on the TME. Most cancer immune cycles were more active in patients with high a VM_Score; for example, the release of cancer cell antigens (step 1), trafficking of immune cells to the tumor (step 4) (recruitment of CD8 T cells, CD4 T cells, macrophages, Th1 cells, NK cells, and DCs), and killing of cancer cells (Step 7) (Figure 5A). These immune cycles were also upregulated, resulting in increased levels of infiltration of the corresponding TIICs (which included CD8 T cells, CD4 T cells, NK cells, Th1 cells, macrophages, and DCs) in the BLCA TME. It was performed in six independent algorithms cross-validation (Figure 5B). The results suggest that those with a high VM_Score phenotype may be more sensitive to ICB because they show an inflammatory phenotype. Our next step was to examine the correlation between the VM_Score and predictors of ICB effectiveness. First, by analyzing the relationship between the VM_Score and TIS, we found that they were positively correlated (Figure 5C). Low VM_Score groups had lower enrichment scores for immune checkpoints (such as, PD-L1, TIGIT, and CTLA-4) and immunotherapy response genes when compared with high VM_Score groups (Figures 5D, E). In the stratified analysis of age, sex, and stage, there was a consistent trend (Supplementary Figures S7–S12).

All results suggested that the VM_Score was correlated with the TME and may be a potential predictor of ICB efficacy in BLCA.

### 3.6 Effect of VM_score for molecular subtypes and therapeutic response

**FIGURE 6:**
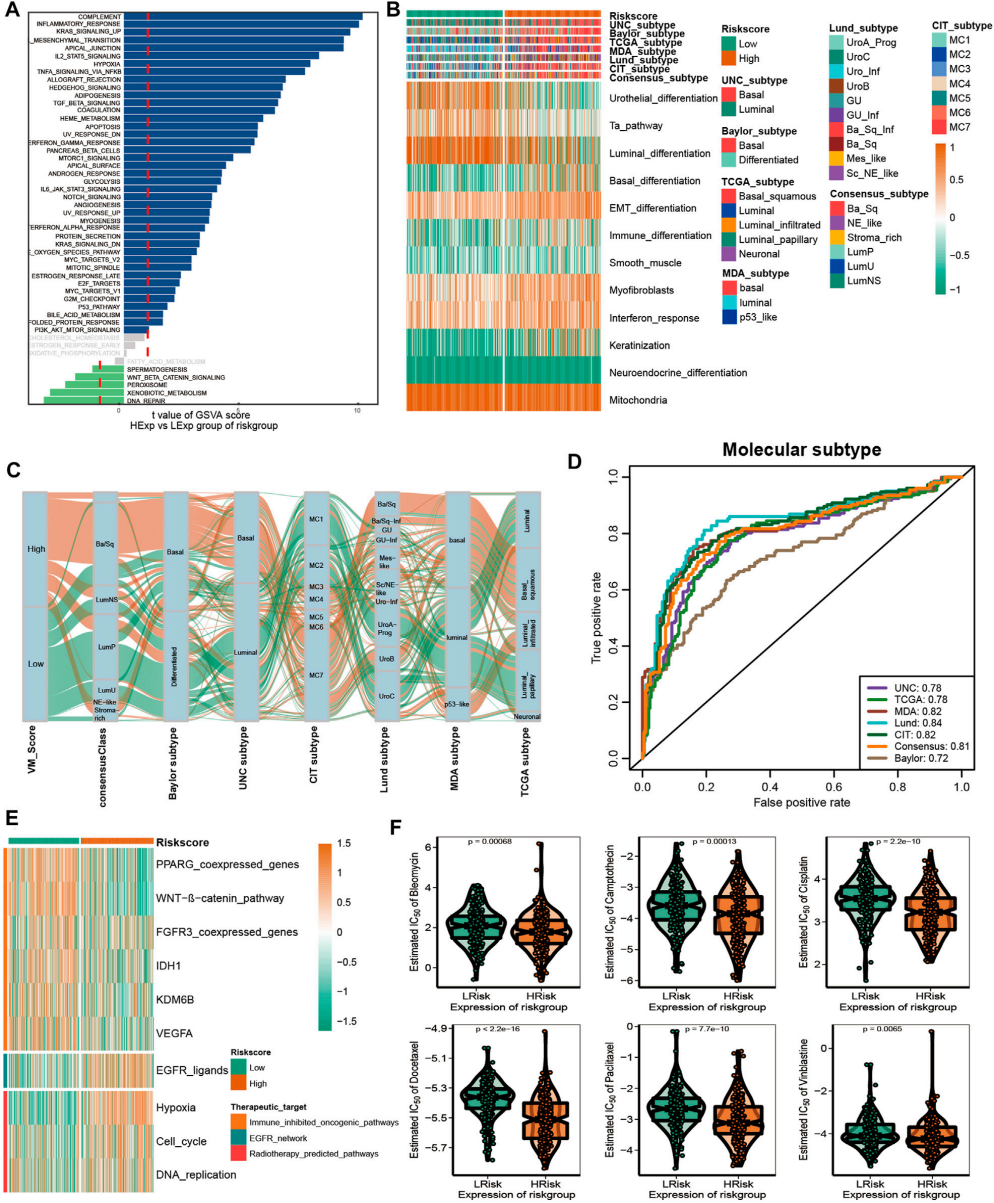
VM_Score effectively predicted molecular subtypes and therapy response. **(A)** Differences in biological function between VM_Score groups. **(B,C)** Relationship between VM_Score and seven classical molecular subtypes. **(D)** Predictive accuracy of VM_Score for molecular subtypes in multiple different algorithms. **(E)** Relationship between VM_Score and enrichment scores of multiple therapeutic signatures. **(F)** Difference on the effects of six chemotherapy drugs.

In recent years, the emergence of molecular subtypes has promoted the development of precise diagnosis and treatment, and the prognosis and treatment response of BLCA can be better predicted through the refinement of molecular subtypes. After consulting the literature, it was found that there are currently seven reported molecular types of BLCA (Kamoun et al., 2020). However, their clinical application is hindered by the designation of molecular subtypes and the complex detection methods required. Initially, we compared the enrichment of several signaling pathways between high-risk and low-risk groups. In addition, the results indicated that there were significant differences between the high and low VM_Score groups with regard to biological functions (Figure 6A). A high-risk group showed a lot of signals related to complement, inflammation, and EMT, while a low-risk group showed a lot of signals related to DNA repair and xenobiotic metabolism. These results suggest that VM-Score may affect the progression of BLCA by regulating the hallmark signaling pathway. In Figures 6B, C, correlations between the VM_Score and classical molecular subtype classification are shown. According to seven molecular classifications, the high-risk group represented the basal subtype of BLCA: TCGA subtype (Robertson et al., 2017), Cartes d’Identité des Tumeurs-Curie (CIT) subtype (Rebouissou et al., 2014), MD Anderson Cancer Center (MDA) subtype (Choi et al., 2014), Lund subtype (Marzouka et al., 2018), University of North Carolina (UNC) subtype (Damrauer et al., 2014), Baylor subtype (Mo et al., 2018), and consensus subtype (Kamoun et al., 2020), which was characterized by basal differentiation, EMT differentiation, immune differentiation, interferon response, and myofibroblasts; as opposed to low VM_Scores, which are characterized by luminal differentiation, urothelial differentiation, and the Ta pathway. The ROC curves showed that the VM_Score had a high prediction accuracy for molecular subtypes, mostly exceeding 0.75 (Figure 6D).

A number of different treatment regimens were tested for their ability to predict their responses to VM_Score. It is possible that patients with high VM_Scores are more susceptible to EGFR-targeted therapy and radiotherapy. A significant VM_Score enrichment was found in the low VM_Score group for several immunosuppressive oncogenic pathways, such as the Wnt–β-catenin network, IDH1, PPARG network, KDM6B, and VEGFA (Figure 6E). The targeting of these oncogenic pathways may provide promising therapeutic opportunities for BLCA patients with low VM_Scores. Furthermore, chemotherapy drugs were more sensitive in patients with a high VM_Score, such as cisplatin, paclitaxel, bleomycin, camptothecin, docetaxel, and vinblastine (Figure 6F).

Overall, the VM_Score may be a cost-effective and simpler alternative to classical molecular subtypes. In addition, the VM_Score can be used to predict how well BLCA patients will respond to several treatments.

### 3.7 Validating roles of VM_score in Xiangya cohort and GSE32894

**FIGURE 7:**
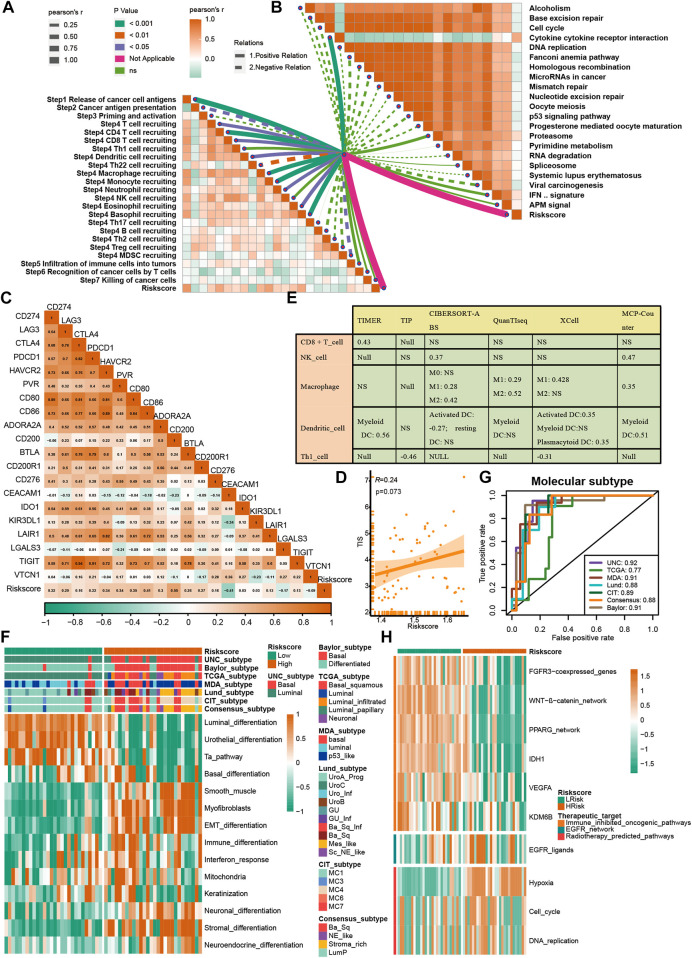
Validation of VM_Score in Xiangya cohort. **(A)** Relationship between VM_Score and activities of cancer immunity cycles. **(B)** Relationship between VM_Score and immunotherapy predicted pathways. **(C)** Correlations between VM_Score and several immune checkpoints. **(D)** Relationship between VM_Score and T cell inflammation score (TIS). **(E)** Relationship between VM_Score and infiltration levels of five tumor-infiltrating immune cells. **(F)** VM_Score accurately stratified the molecular subtypes in seven different algorithms. **(G)** Accuracy of VM_Score in predicting molecular subtypes in seven different algorithms. **(H)** Relationship between VM_Score and enrichment scores of several therapeutic signatures.

**FIGURE 8:**
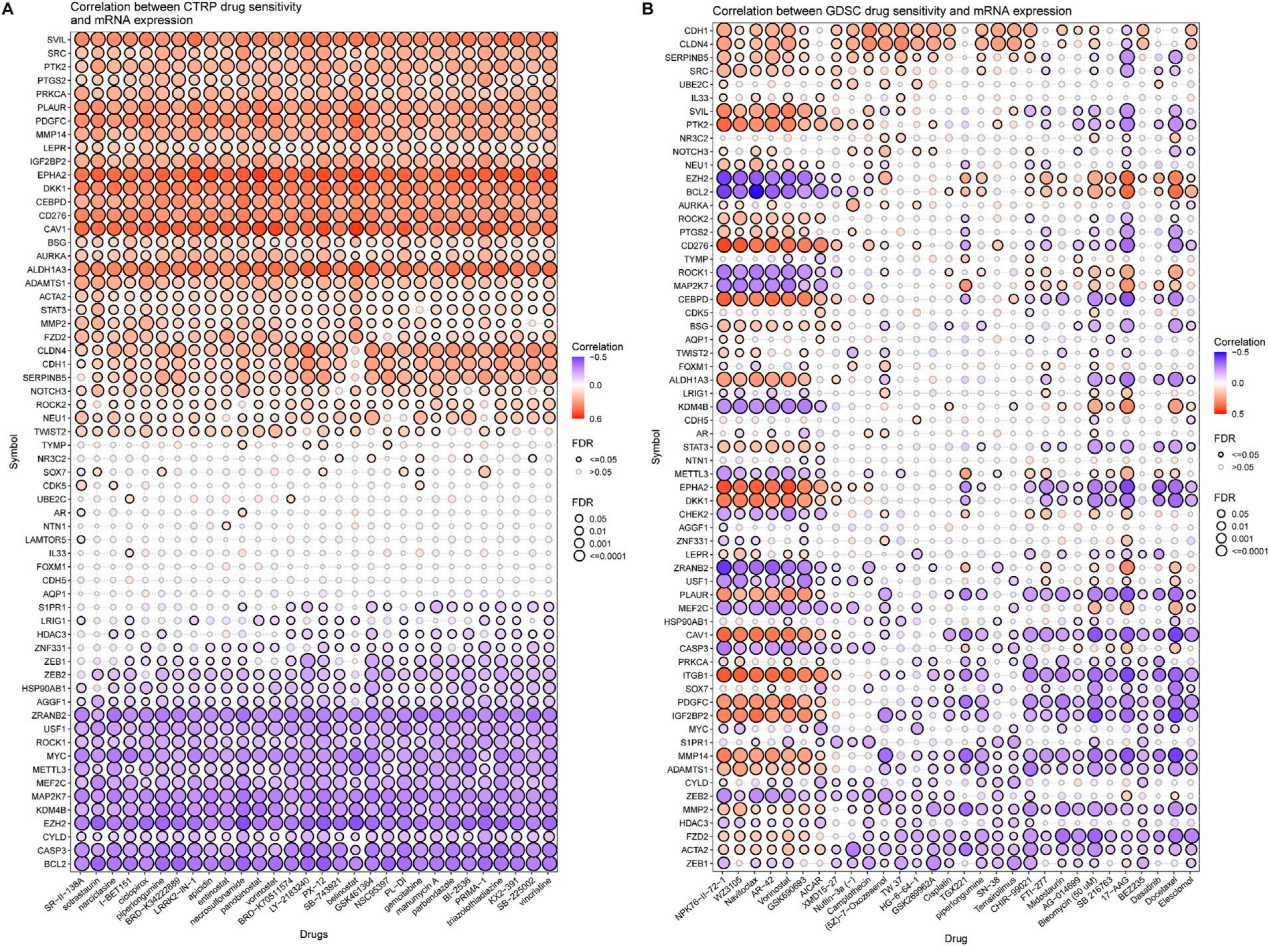
Correlation analysis between VM-related DEGs and drug sensitivity (top30 drugs in GDSC and CTRP). **(A, B)** Bubble chart shows the correlation analysis between VM-related genes and drug susceptibility. Red indicates positive correlation and blue indicates negative correlation. The darker the color, the higher is the correlation index. Bubble size indicates FDR.

A further validation of the capability of the VM_Score to predict the immune status, molecular subtype, and treatment response has been performed in our cohort (Xiangya cohort). Multiple anticancer immune cycles and infiltration levels of CD8 T cells, Th1 cells, DC, NK cells, and macrophages were positively correlated with the VM_Score in six independent algorithms (Figures 7A, E). Furthermore, the VM_Score was significantly correlated with the enrichment scores of immune response–related signatures, TIS, and ICB (Figures 7B–D). Data from this study have suggested that the VM_Score is an effective tool for stratifying BLCA immunophenotypes. Additionally, the VM_Score can accurately predict classical molecular subtypes with AUCs usually around 0.90, except in the TCGA data set (AUC = 0.77) (Figures 7F, G). There is a promising correlation between VM_Score and clinical response to radiotherapy, as well as several targeted therapies. It appears that patients in the high-risk group may benefit more from radiotherapy and EGFR-targeted therapies. A targeted therapy, such as blocking the Wnt–β-catenin network or the FGFR3 network, may be better suited for patients at low risk (Figure 7H). The abovementioned results were all validated in GSE32894 (Supplementary Figure S13). In addition, we looked at the correlation between 66 VM-related DEGs and sensitivity to multiple drugs, and we found that CYLD, MYC, and ZEB1 were negatively related to most drugs, while CDH1, CLDN4, and SERPINB5 were positively associated with major drug sensitivity (Figure 8, Supplementary Figures S13, S15).

## 4 Discussion

There are increasing numbers of studies showing that VM plays a critical role in the regulation of tumors; however, the role of VM in BLCA is unclear. In this study, we developed a new systemic indicator of molecular VM–related genes mediated by molecular subtypes based on the Xiangya cohort and additional external BLCA cohorts using a random forest algorithm. A correlation was then done between the VM_Score and classical molecular subtypes, clinical outcomes, immunophenotypes, and treatment options for BLCA. With the VM_Score, it is possible to predict classical molecular subtypes, immunophenotypes, prognosis, and therapeutic potential of BLCA with high accuracy. VM_Scores of high levels indicate a more anticancer immune response but a worse prognosis due to a more basal and inflammatory phenotype. the VM_Score was also associated with low sensitivity to antiangiogenic and targeted therapies that target the FGFR3, β-catenin, and PPAR-γ pathways but are associated with high sensitivity to cancer immunotherapy, neoadjuvant chemotherapy, and radiotherapy. A number of aspects of BLCA biology were reflected in the VM_Score, providing new insights into precision medicine. Additionally, the VM_Score may be used as an indicator of pan-cancer immunotherapy response and prognosis.

We collected molecular VM-related genes through keyword search, performed differential analysis in BLCA and the adjacent tissues to screen out 66 differentially expressed genes, and constructed the VM_Score by the random forest method. Then, we validated it in multiple data sets such as our own Xiangya cohort, and the results showed that the VM_Score can predict the clinical outcome, molecular subtype, and treatment response of BLCA and was related to the inflammatory TME. VM affects tumor progression and survival prognosis. Studies have found that VM was significantly associated with the survival and prognosis of various tumors (Cao et al., 2013). In this study, the KEGG and GO enrichment analyses of VM-related differential genes showed that they were mainly enriched in cell junction assembly, tissue remodeling, regulation of anoikis, regulation of peptidase activity, and focal adhesion. These pathways were closely related to tumor invasion and migration. In addition, through analyzing the differences among 50 signaling pathways between the VM_Score groups, it was found that EMT was most abundant in the high VM_Score group and DNA repair was more abundant in the low-risk group. EMT and DNA damage are involved in key steps in tumorigenesis and development (Aiello and Kang, 2019; Srinivas et al., 2019). Genomic instability promotes tumor progression (Andor et al., 2017). Through mutation analysis of VM-related DEGs, it was found that most genes have high mutation frequencies in various tumors such as UCEC, SKCM, and BLCA. The gene copy number variation was mainly heterozygous amplification and deletion. The study concluded that VM may play a significant role in the occurrence and development of tumors.

The VM_Score predicts molecular subtype and treatment response in BLCA. Researchers found that the higher the VM_Score, the higher is the tumor stage, and worse is the survival prognosis for BLCA patients. In addition, the opposite was the case with low VM_Scores. The correlation between VM-related DEGs and the sensitivity of various drugs was analyzed in CTRP and GDSC databases, and many genes were found to be closely related to most drugs. There was a greater sensitivity to chemotherapy drugs such as cisplatin, paclitaxel, bleomycin, docetaxel, camptothecin, and vinblastine, and radiotherapy and EGFR-targeted therapy in the high VM_Score group, whereas drugs that target oncogenic pathways such as Wnt–β-catenin network, PPARG network, IDH1, KDM6B, and VEGFA may be more effective for the low VM_Score group. Molecular subtype has greatly facilitated precision medicine. In recent years, a variety of molecular typing has been reported, but they have not been used in clinical practice due to factors such as complex detection methods. According to this study, the low VM_Score group mainly represented luminal types, and the high VM_Score group represented the basal type, and the prediction was more accurate. Therefore, the VM_Score is a more reliable prediction tool, which is of great help in accurate diagnosis and treatment by representing different molecular subtypes.

VM correlated with the tumor microenvironment and guided immunotherapy. Studies have shown that vasculogenic mimicry structures support the recruitment of monocytes (Tan et al., 2022). In addition, the KEGG and GO enrichment analyses of molecular VM-related DEGs found that they were significantly enriched in focal adhesion and regulation of anoikis. There was a significant association between a novel focal adhesion-related gene signature and tumor grade, tumor stage, immune scores, and immune infiltrate types, determined by Lin et al. (2021). It was discovered by Fanfone et al. (2022) that anoikis-resistant mechanically stressed cancer cells were more motile and had a better immune response against natural killer cells. The enrichment analysis of the high and low VM_Score groups showed that the complement and inflammatory responses had more abundant signals in the high VM_Score groups. In addition, according to this study, the VM_Score had a positive correlation with anti-cancer immune cycle and TIIC, suggesting that the VM_Score could be related to an inflammatory immune microenvironment, and patients with high VM_Scores had higher anti-cancer immunity. The vascular endothelial growth factor receptor-1 (VEGFR-1) is a membrane receptor which plays a crucial role in melanoma vasculogenic mimicry. VEGFR-1 mediated signaling as an effective target for reducing pre-tumor macrophage tumor invasion and improving ICI immunotherapy efficacy (Lacal et al., 2020). Myeloid-derived suppressor cells' (MDSCs) migration and differentiation are enhanced by VEGF signaling. VEGF-a knockdown in tumor cells resulted in decreased infiltration of MDSC and increased infiltration of CD8 T cells (Horikawa et al., 2017). Antiangiogenic therapy by vaccination or adoptive cell transfer a few days before immunotherapy increases the accumulation of T cells within the tumor, and thus improves the anti-cancer efficacy, when compared with vaccination alone (Fukumura et al., 2018). It is well known that ICB has achieved good results in tumor immunotherapy. In addition, the VM_Score was positively correlated with TIS and immune checkpoint scores (such as CD274, CTLA4, PDCD1, TIGIT, and LAG3), as well as the immunotherapy response–related gene signature enrichment scores, suggesting that the TMEs in the low VM_Score group had fewer immunotherapy target points and the ICB treatment effect is not ideal. By analyzing the methylation and expression levels of molecular VM-related DEGs, we found that methylation modification greatly affected the expression of VM-related DEGs. We can reverse the TME by changing the modification pattern of genes in the low VM_Score group, turning “cold tumors” into “hot tumors.” The VM_Score can predict the therapeutic response to ICB and closely relate to the TME, thus providing a new target for ICB combined treatments.

We recognize that this study has certain limitations. Firstly, this study employed bioinformatics analysis, and the accuracy of the prediction is slightly lower. Although these results have been repeatedly validated in multiple public cohorts and our own Xiangya cohort, we still have to conduct *in vivo* and *in vitro* studies on the relevant mechanisms of VM. Secondly, there is a need for prospective clinical trials to validate the clinical value of the VM_Score. Thirdly, the optimal VM_Score cutoff value was not determined.

## 5 Conclusion

The VM_Score developed and validated by our group can successfully predict clinical characteristics, TME characteristics, and survival outcomes of BLCA. The VM_Score was a reliable and effective biomarker and tool for predicting patients’ clinical responses to several therapies. It is possible that patients with a high VM_Score will respond more favorably to immunotherapy, chemotherapy, radiotherapy, and EGFR-targeted therapies. However, patients with low-risk scores may benefit from a number of targeted therapies, such as blocking PPARG, Wnt–β-catenin, and FGFR3.

## Data Availability

The data sets presented in this study can be found in online repositories. The names of the repository/repositories and accession number(s) can be found in the article/Supplementary Material.
