# Supplementary material for: Molecular vasculogenic mimicry–Related signatures predict clinical outcomes and therapeutic responses in bladder cancer: Results from real-world cohorts
Source: Front Pharmacol. 2023 Apr 24;14:1163115. doi: 10.3389/fphar.2023.1163115 (PMC10184144; doi:10.3389/fphar.2023.1163115)

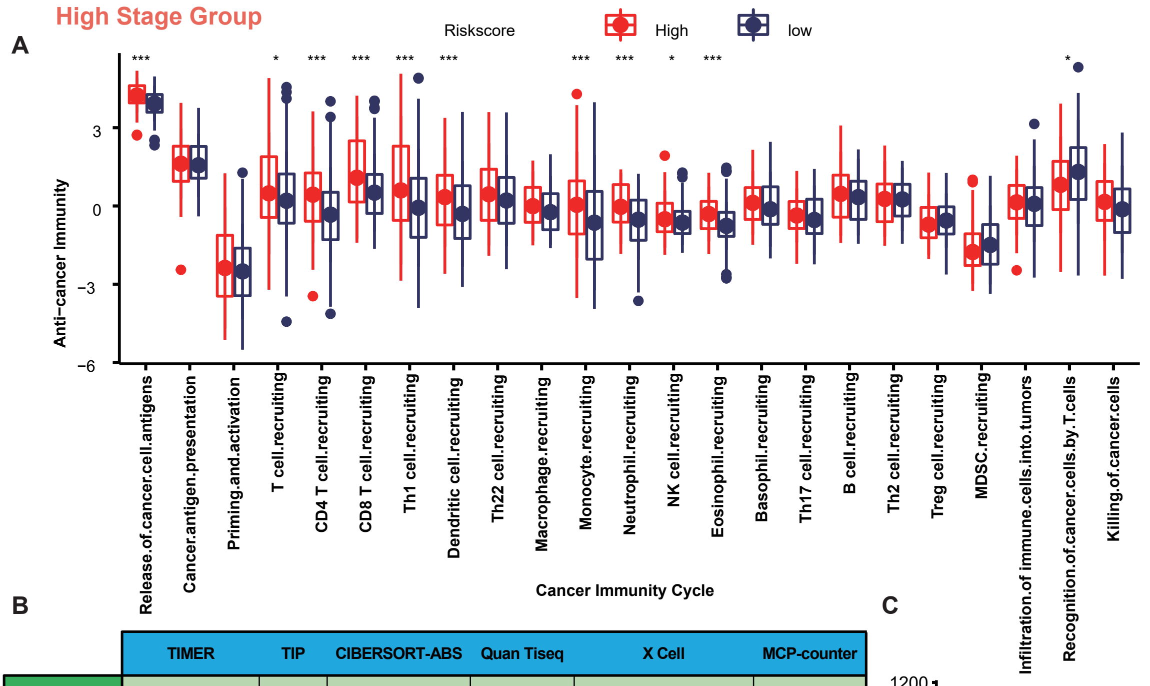

B

	TIMER	TIP	CIBERSORT-ABS	Quan Tiseq	X Cell	MCP-counter
CD8 + T_cell	0.36	Null	NS	NS	NS	NS
NK_cell	Null	NS	Activated :0.21	-0.14	NS	0.22
Macrophage	0.13	Null	M0: 0.18<br>M1: 0.18<br>M2: 0.29	M1: 0.32<br>M2: 0.26	M1: 0.24<br>M2: 0.16	0.41
Dendritic cell	Myeloid DC: 0.40	NS	NS	-0.19	Activated DC: 0.26<br>Myeloid DC:0.19<br>Plasmacytoid DC: 0.26	Myeloid DC:0.31
Th1_cell	Null	NS	Null	Null	NS	Null

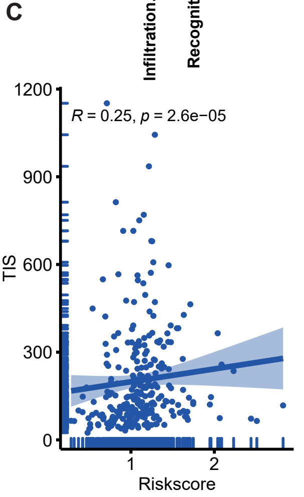

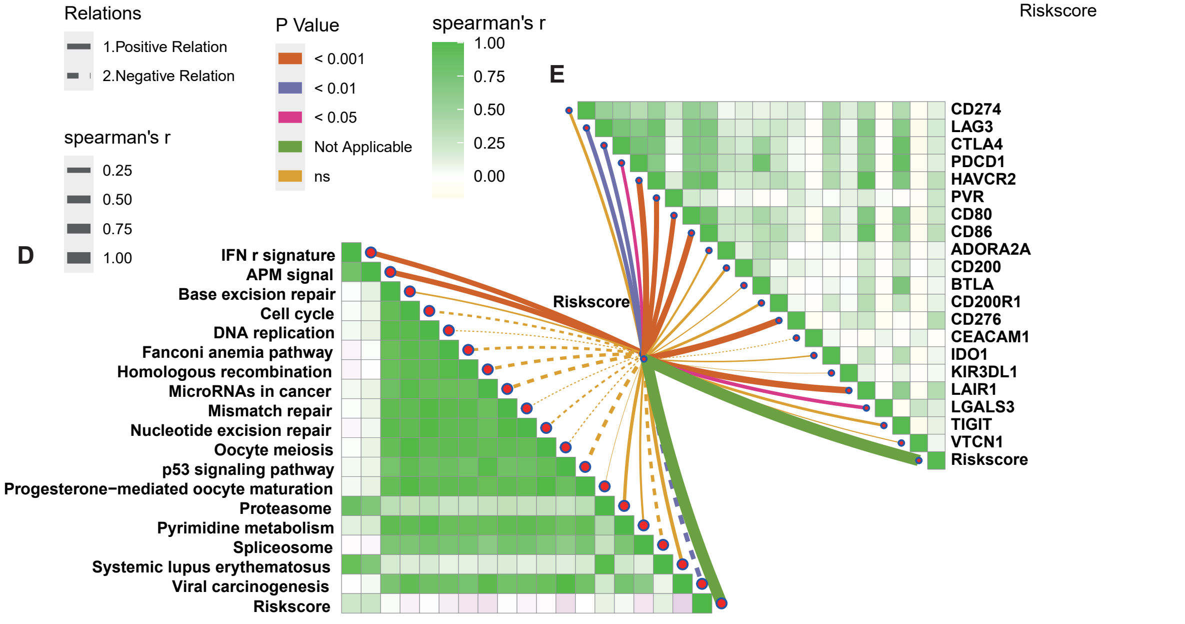

Supplement: Supplementary file 1 [file DataSheet7.PDF]
