# Supplementary figures and images for: Molecular vasculogenic mimicry–Related signatures predict clinical outcomes and therapeutic responses in bladder cancer: Results from real-world cohorts
Source: Front Pharmacol. 2023 Apr 24;14:1163115. doi: 10.3389/fphar.2023.1163115 (PMC10184144; doi:10.3389/fphar.2023.1163115)

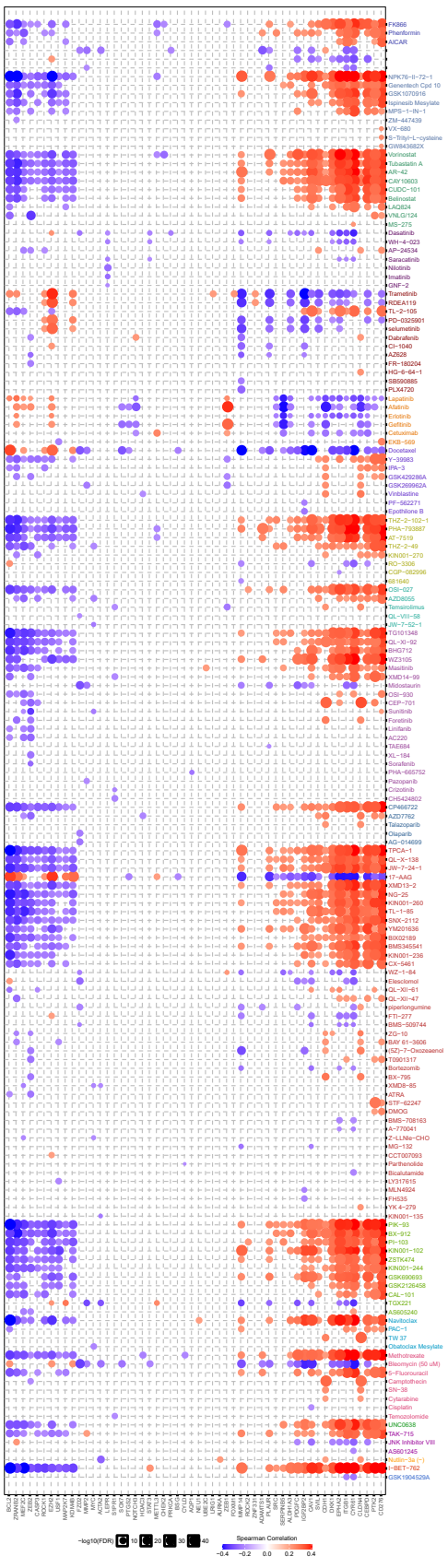

Supplement: Supplementary file 2 [file DataSheet13.PDF]

### CNV percentage in each cancer

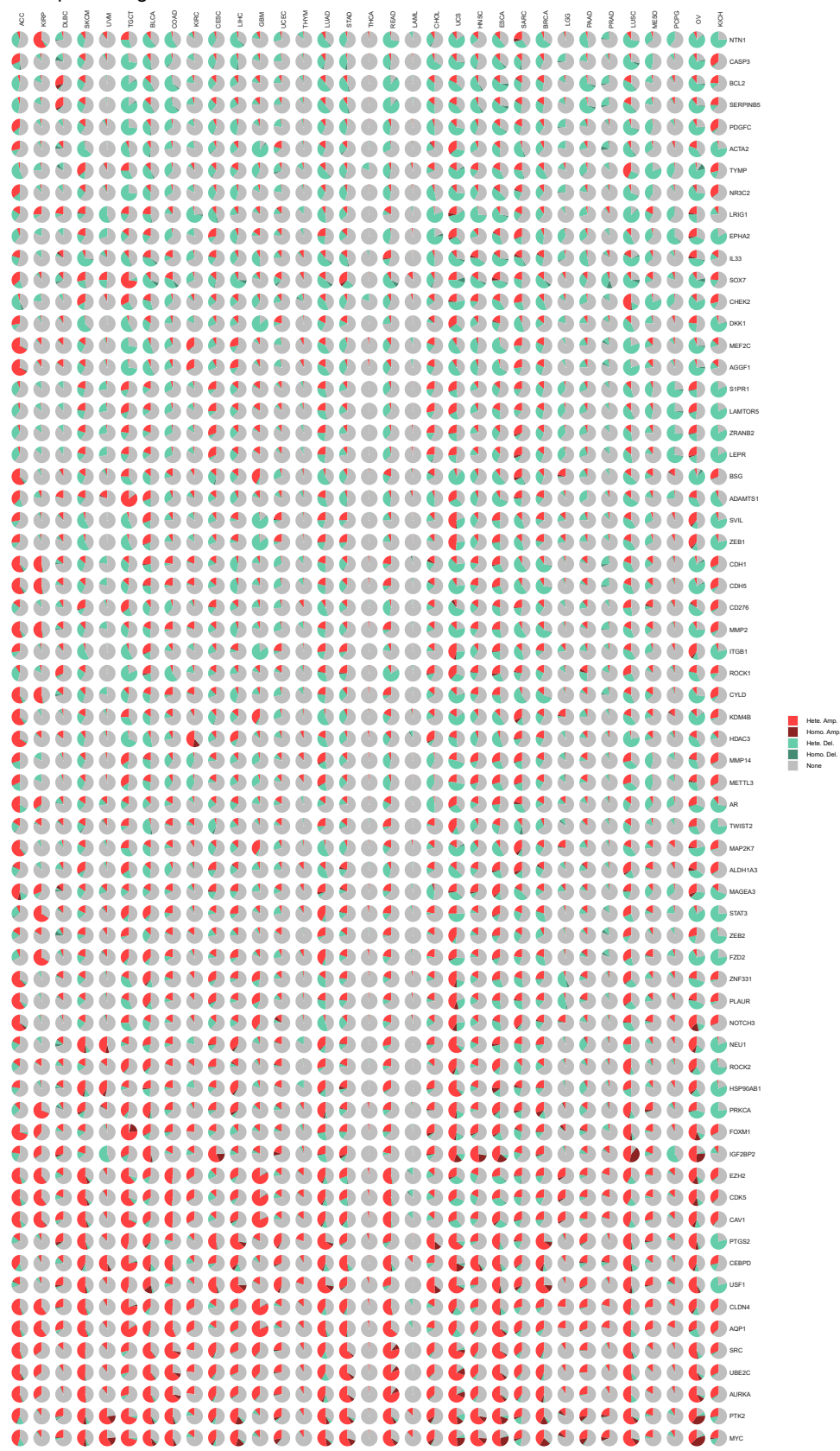

Supplement: Supplementary file 3 [file DataSheet2.PDF]

Correlations of CNV with mRNA expression

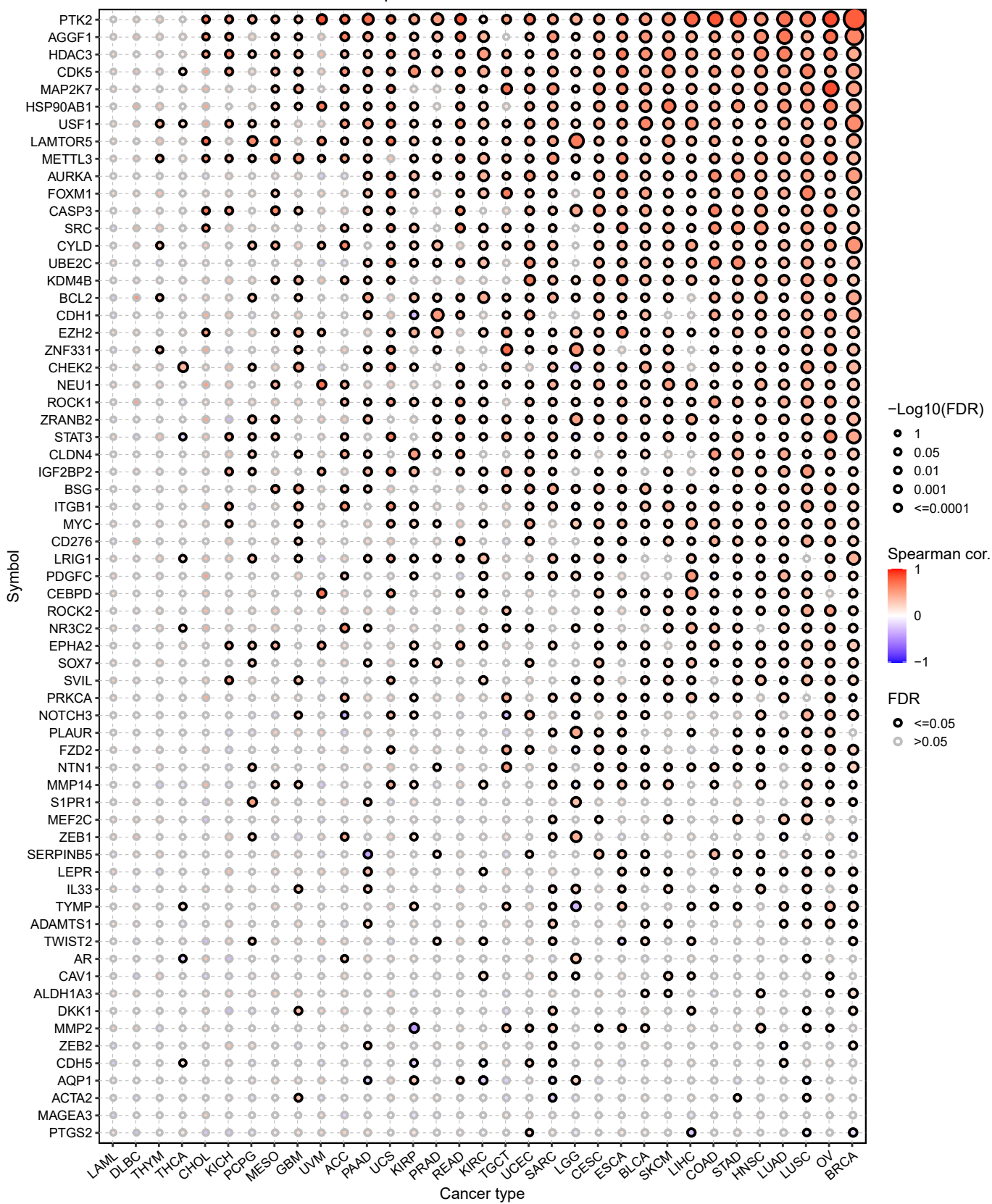

Supplement: Supplementary file 5 [file DataSheet4.PDF]

A

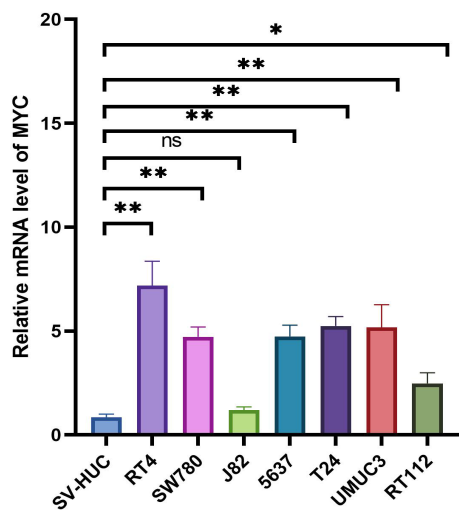

B

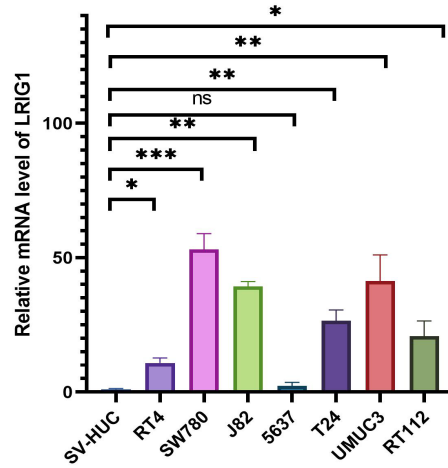

C

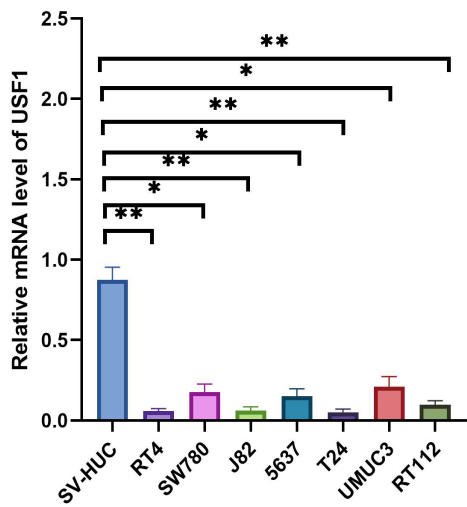

D

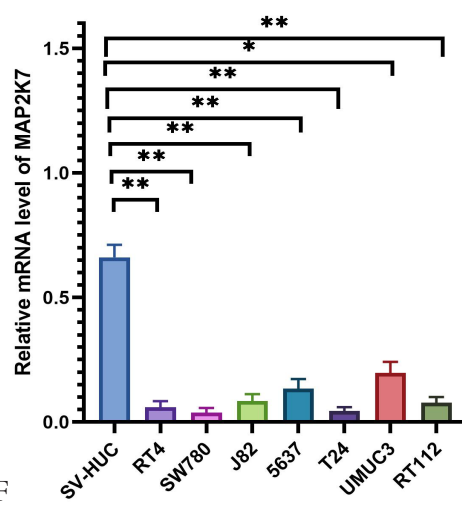

E

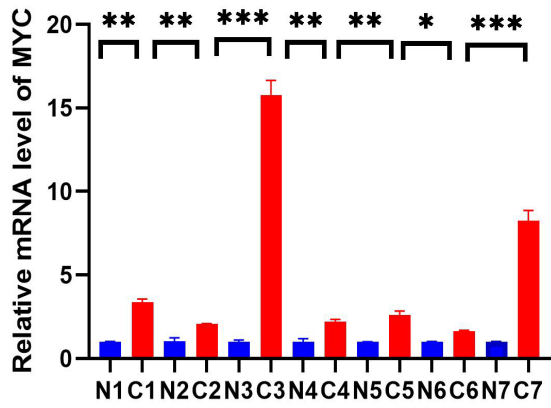

F

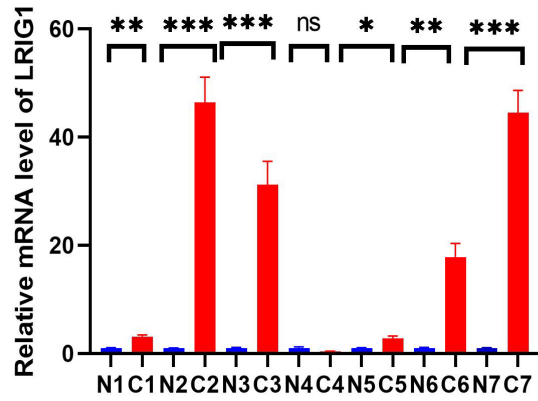

G

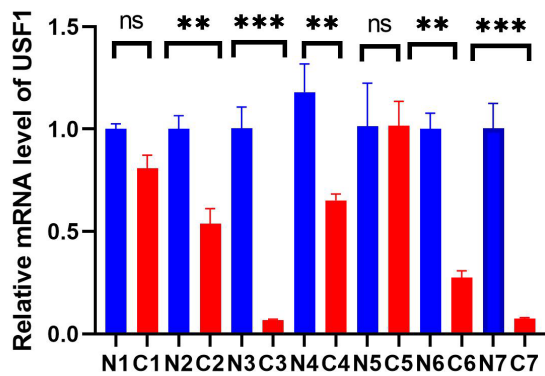

H

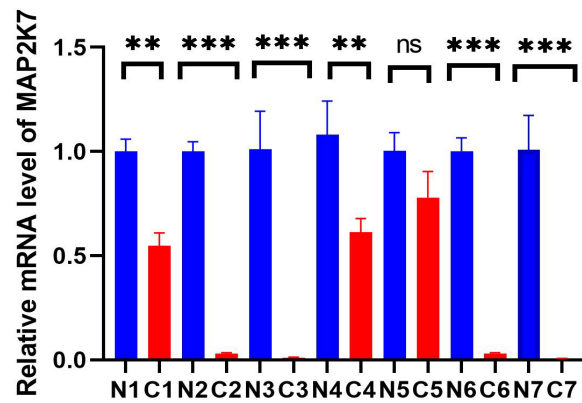

Supplement: Supplementary file 7 [file DataSheet14.PDF]

# GSE32894

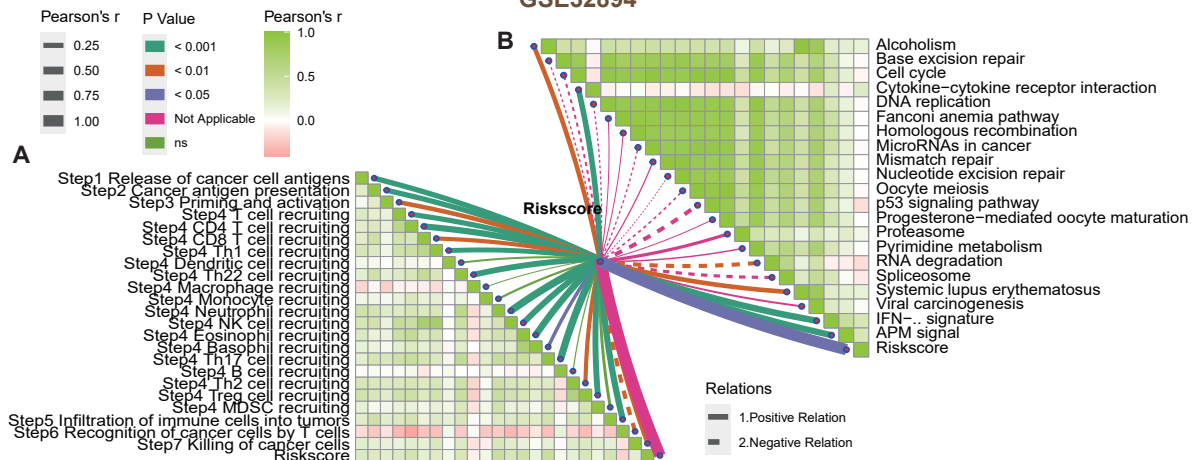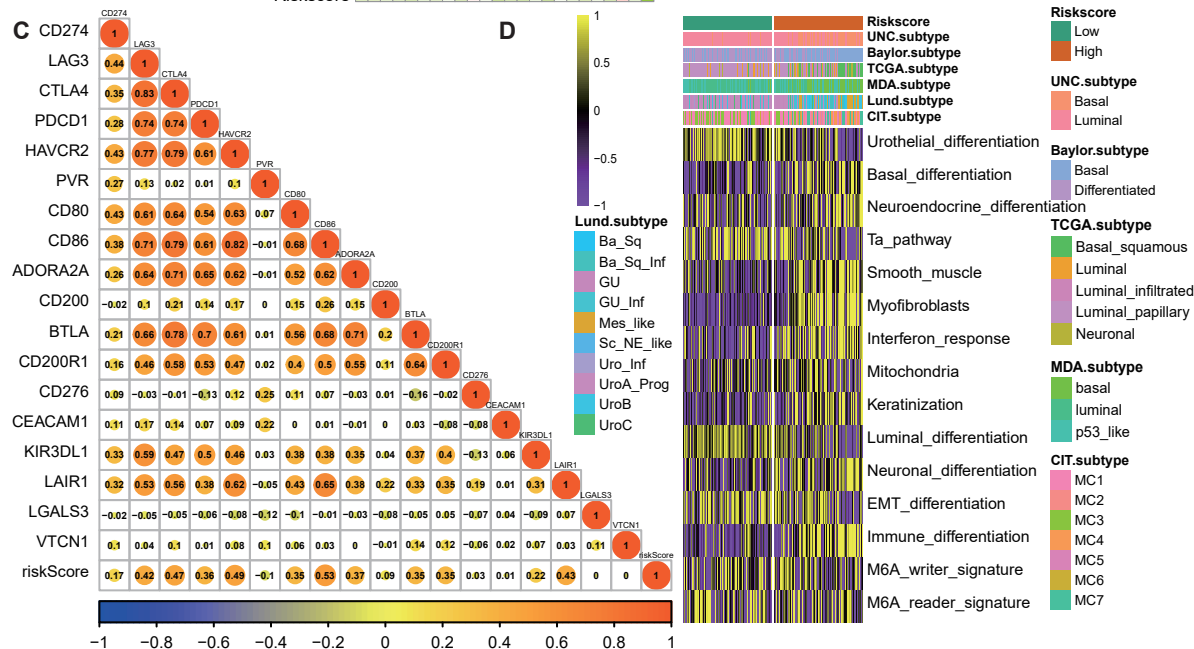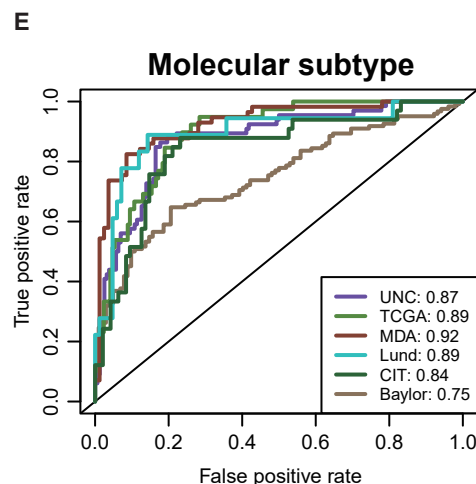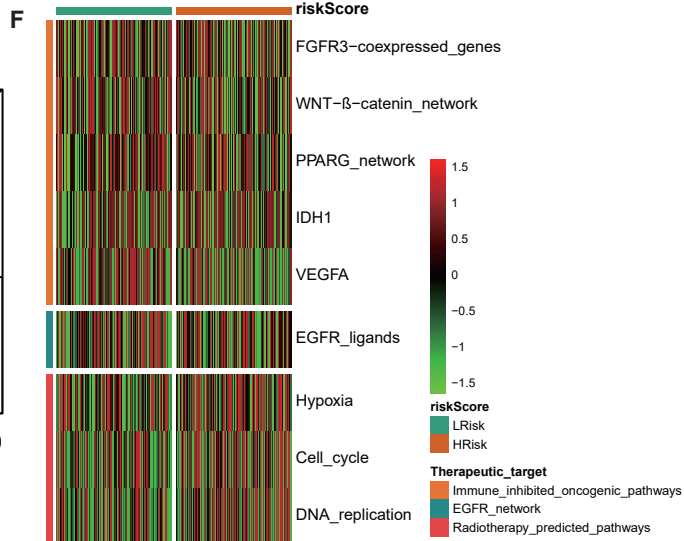

Supplement: Supplementary file 10 [file DataSheet11.PDF]

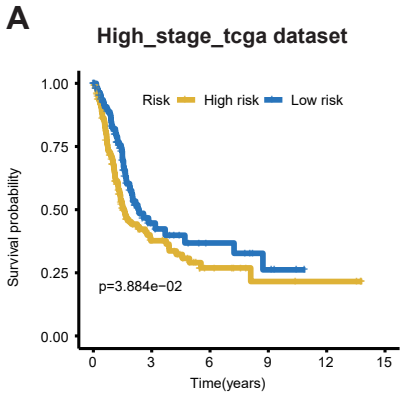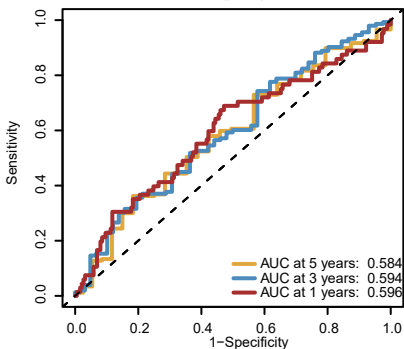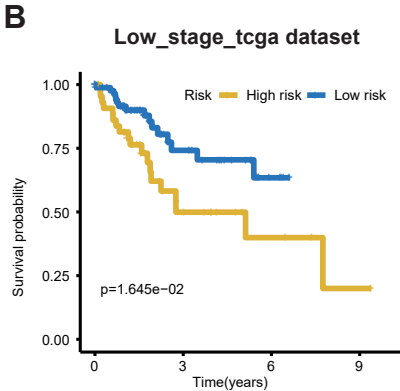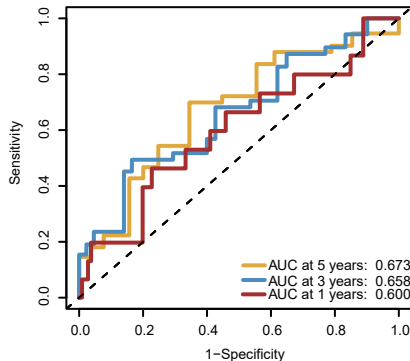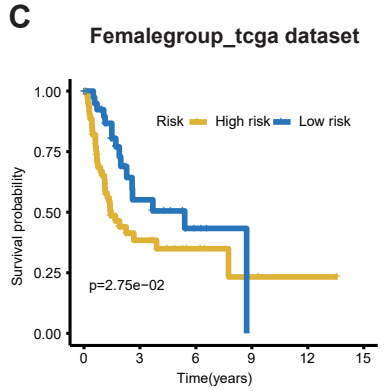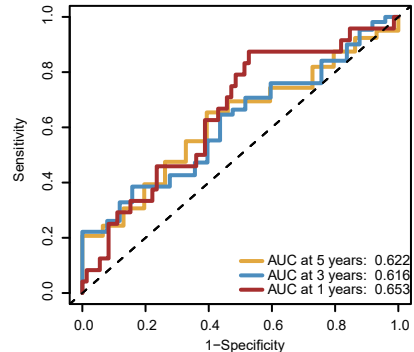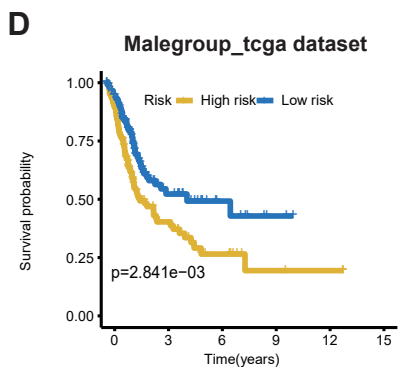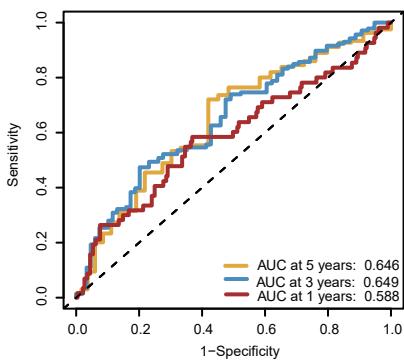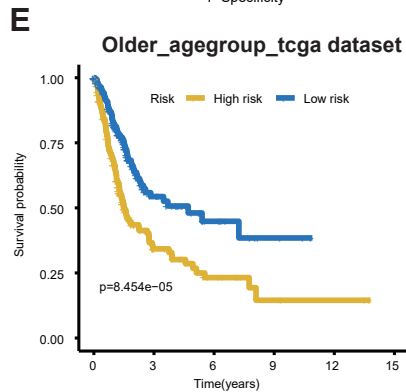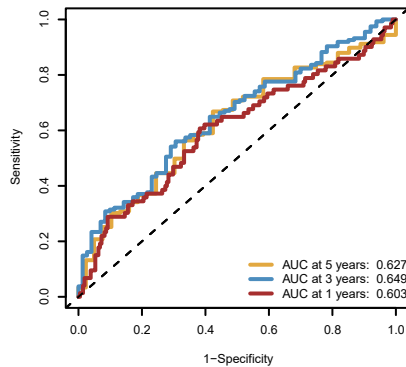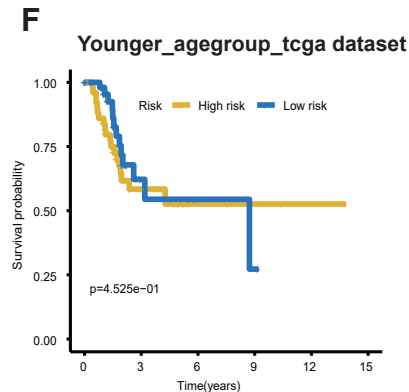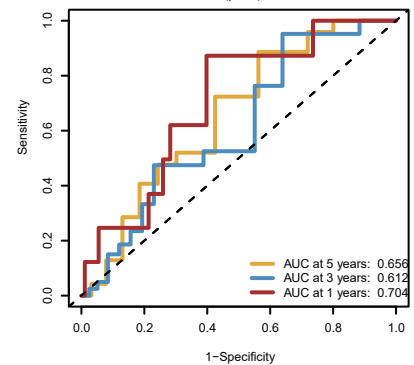

Supplement: Supplementary file 11 [file Image2.PDF]

Correlation between methylation and mRNA expression

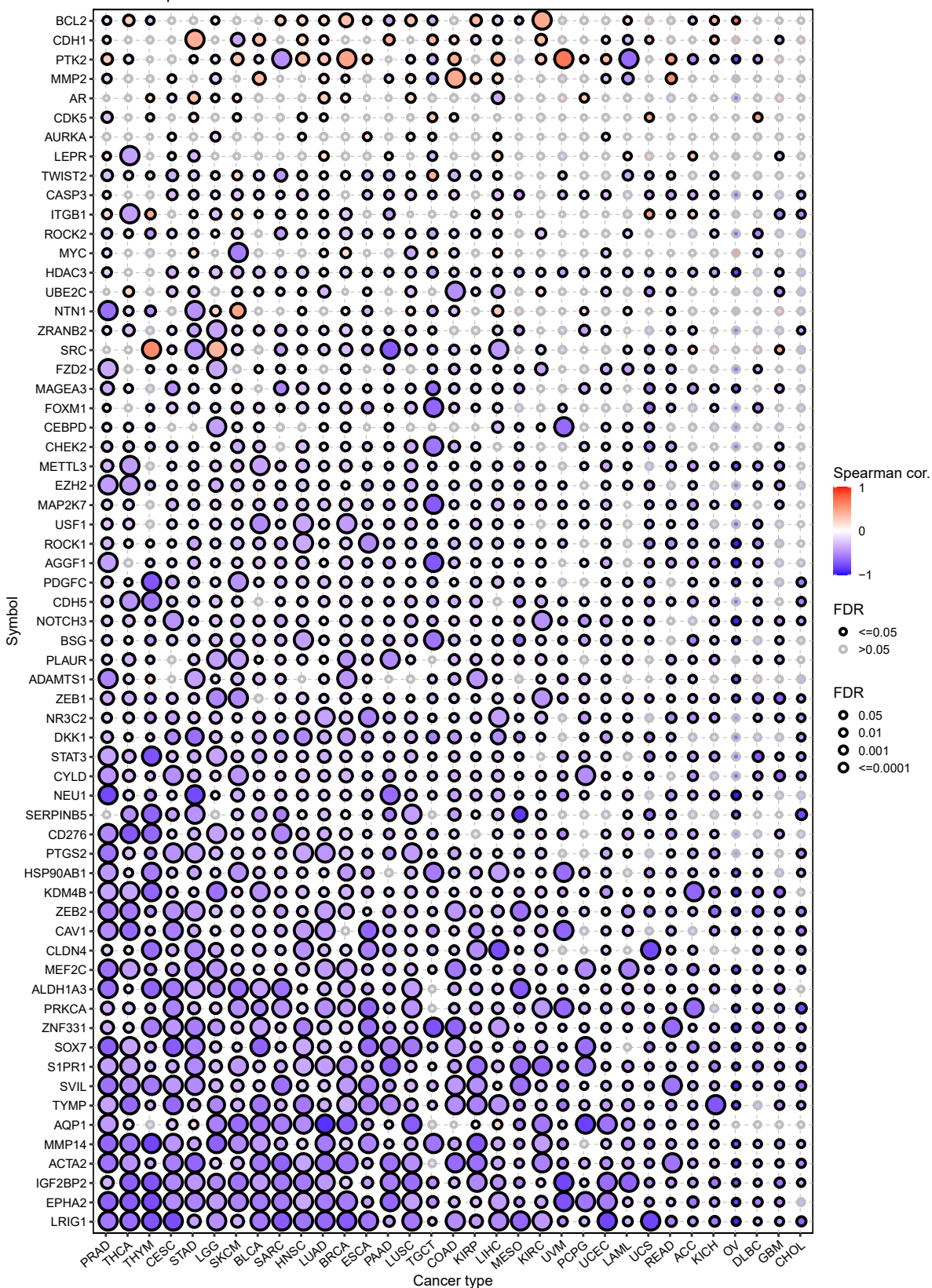

Supplement: Supplementary file 12 [file DataSheet3.PDF]

# Female Group

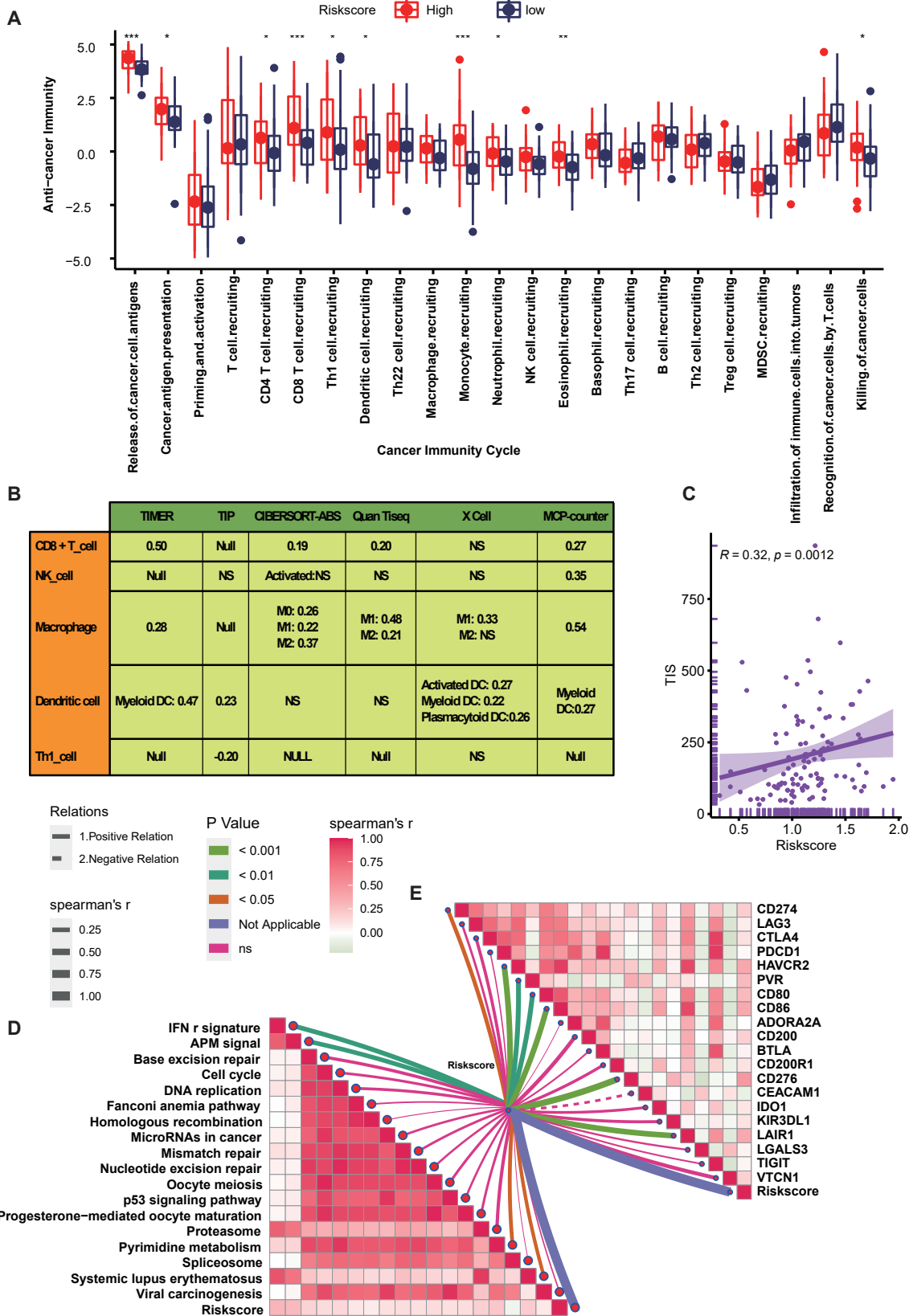

Supplement: Supplementary file 14 [file DataSheet5.PDF]

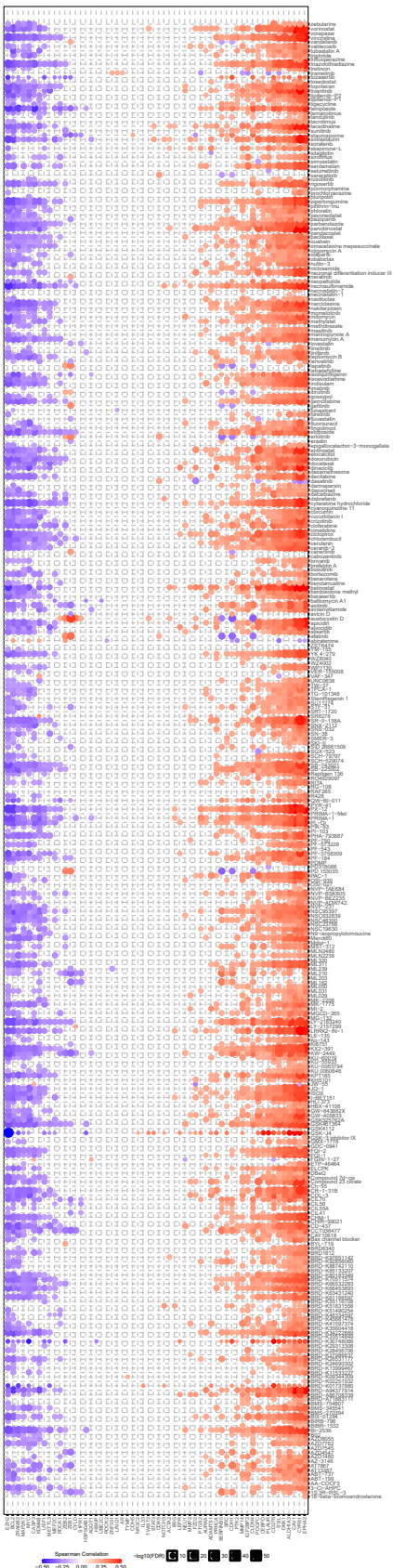

Supplement: Supplementary file 16 [file DataSheet12.PDF]

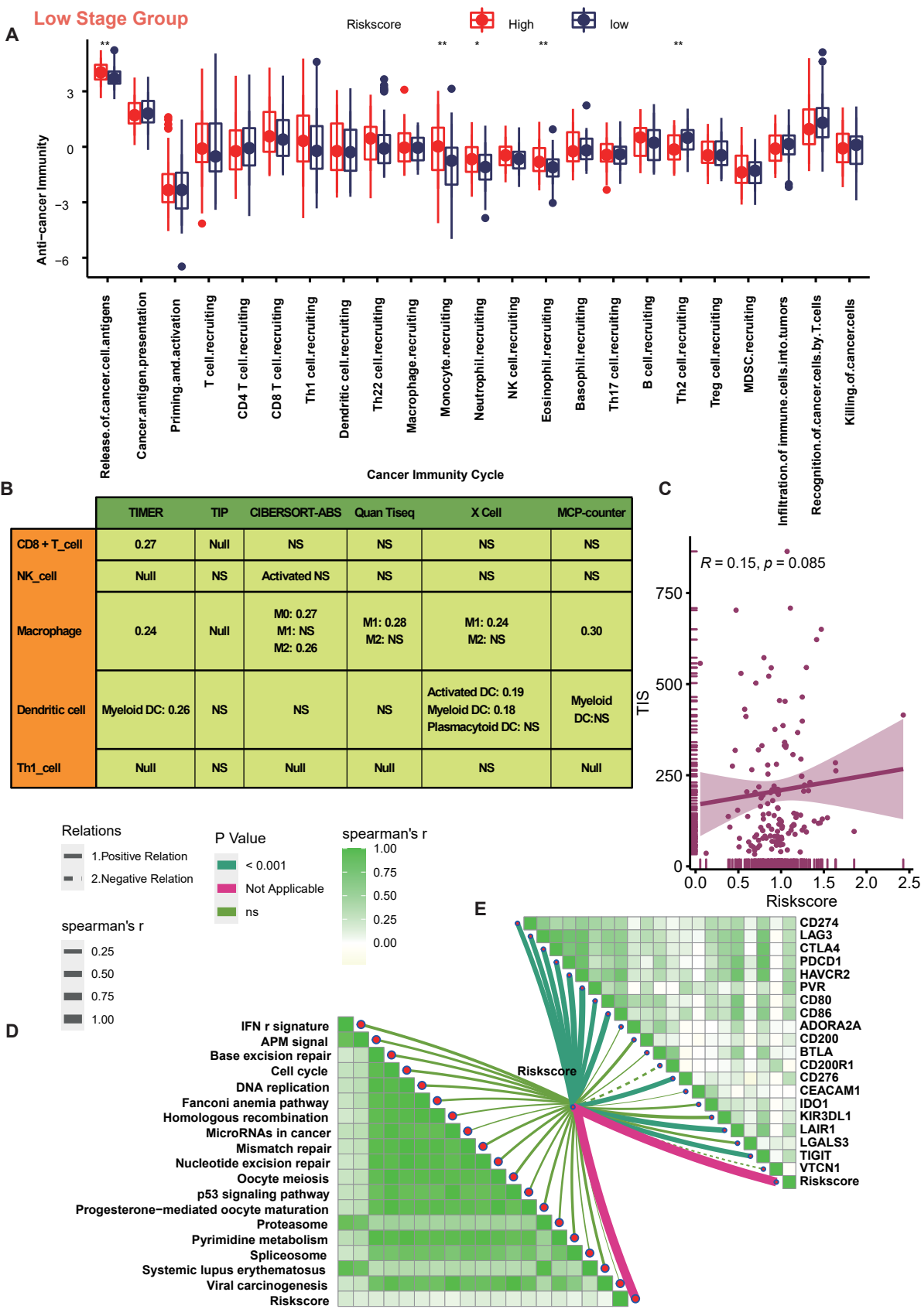

Supplement: Supplementary file 17 [file DataSheet8.PDF]

# Younger Group

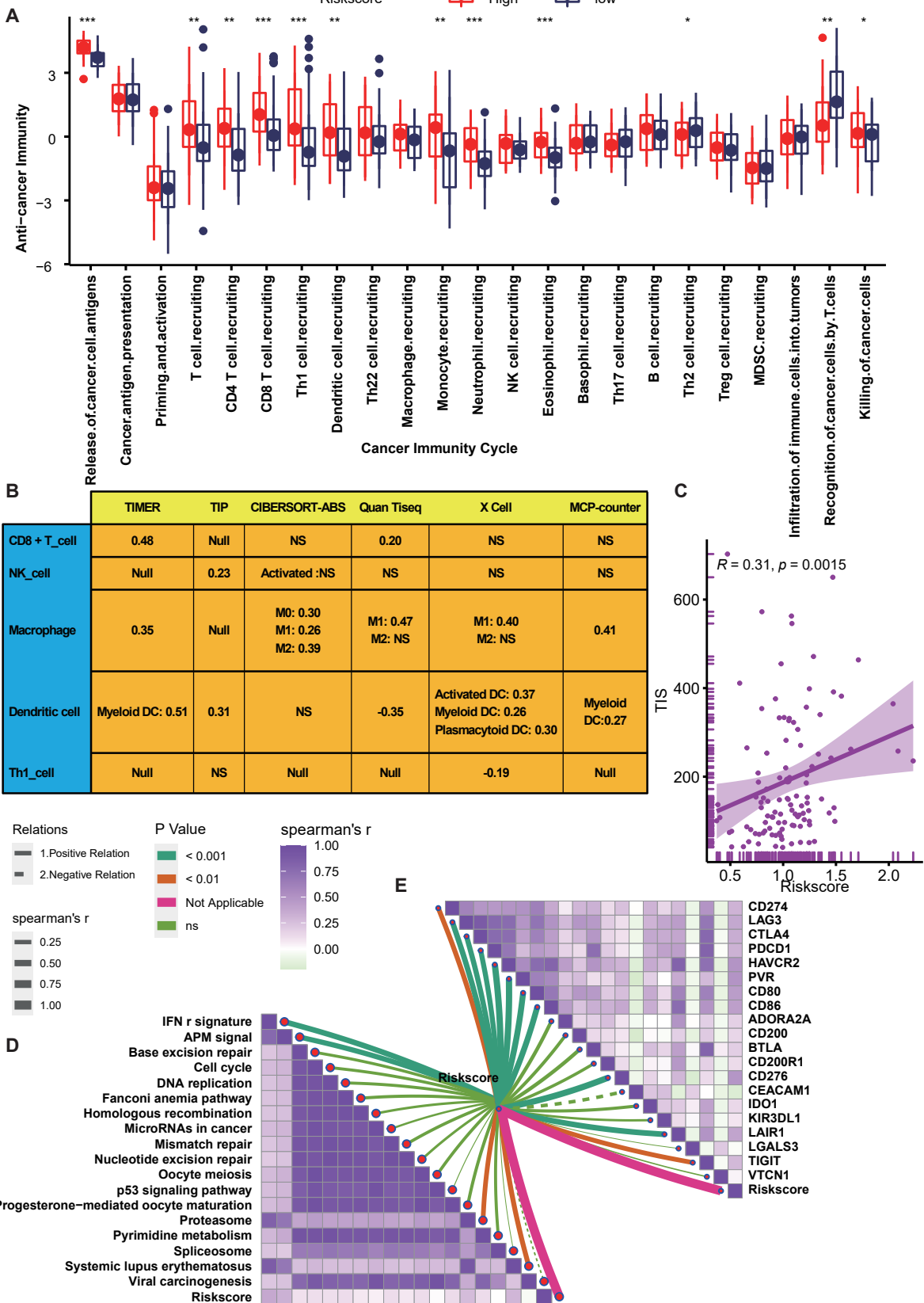

Supplement: Supplementary file 18 [file DataSheet10.PDF]

# GSE13507

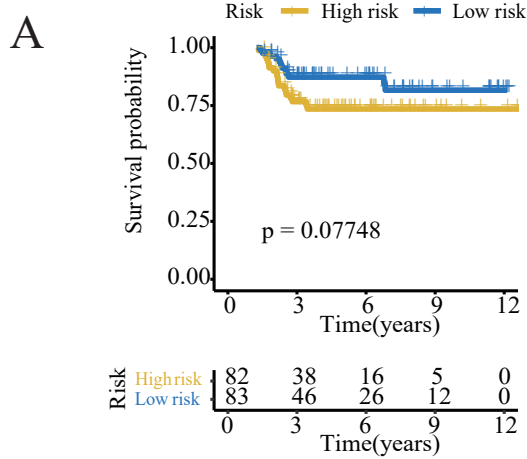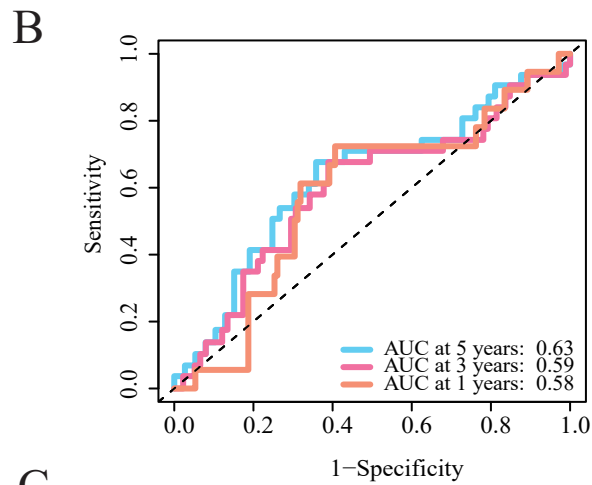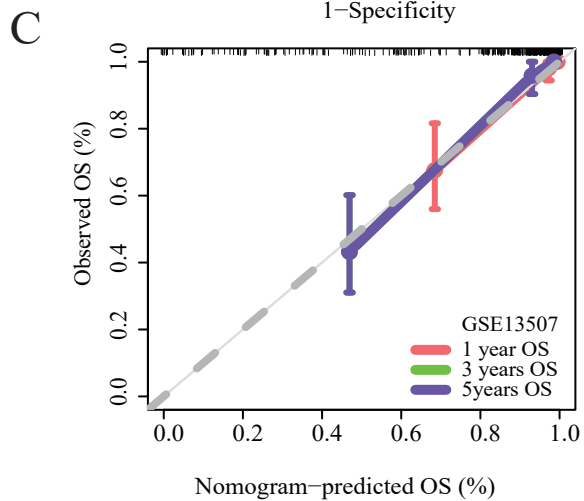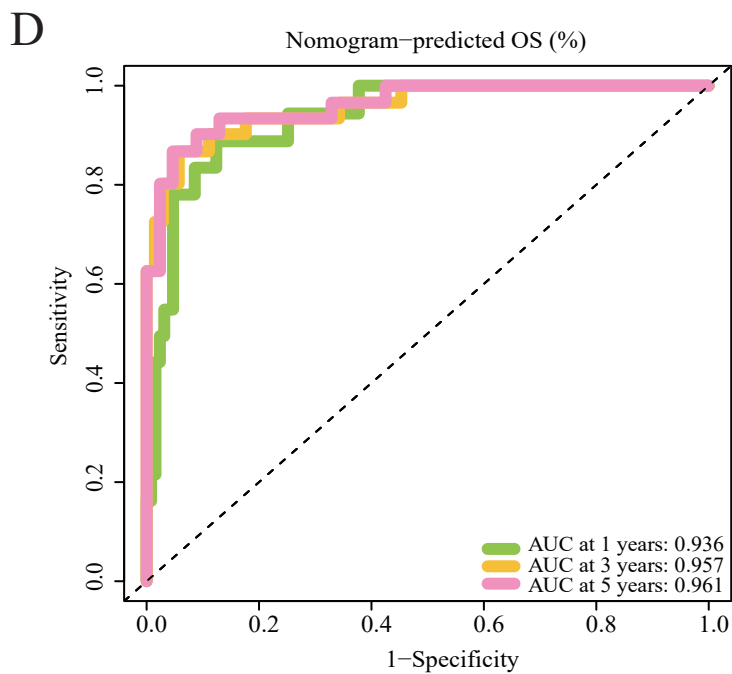

# GSE48075

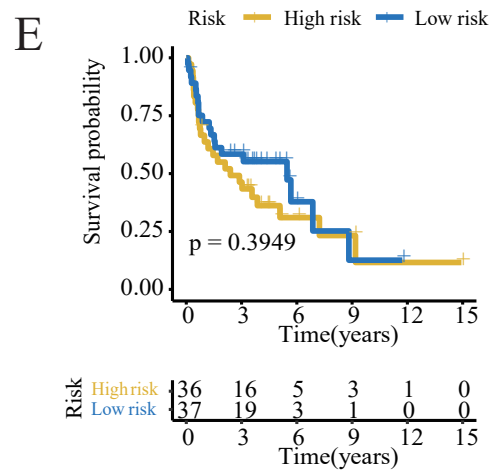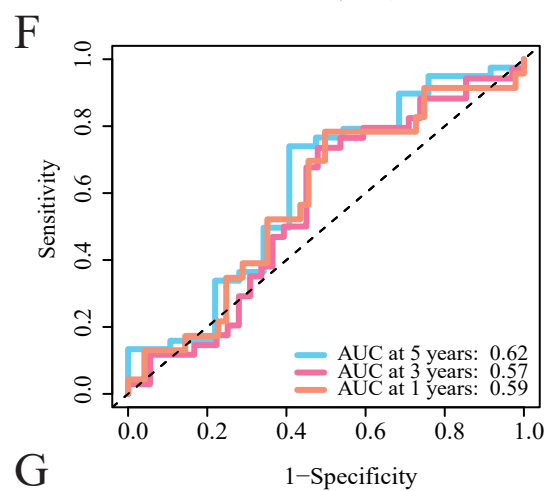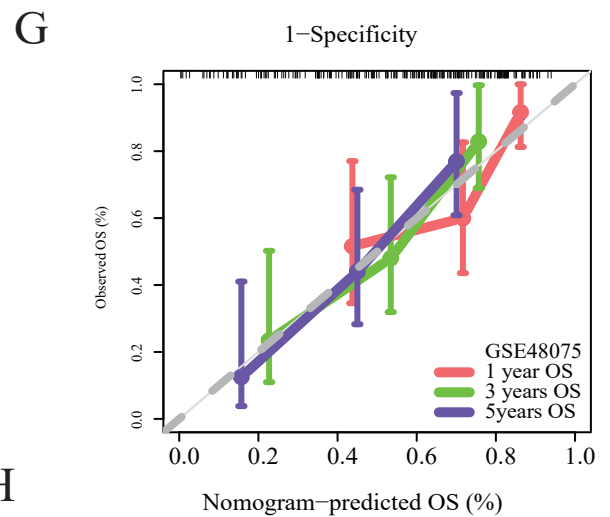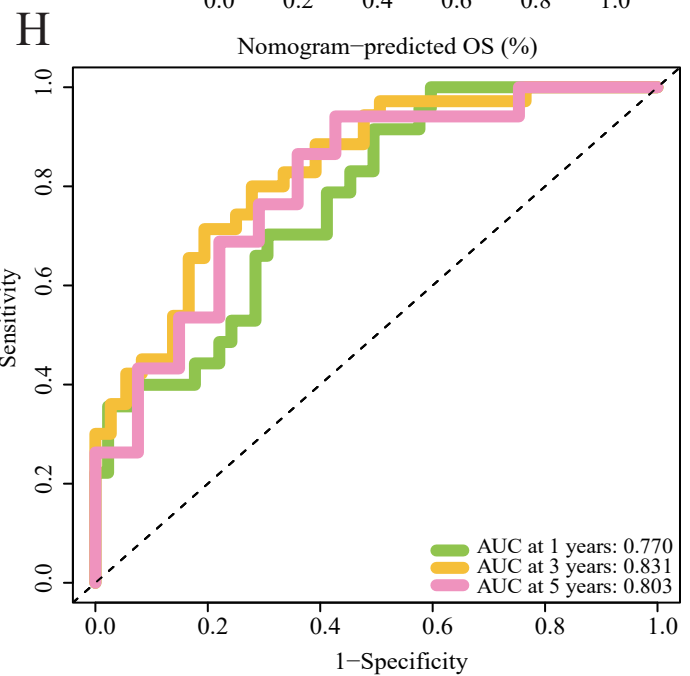

Supplement: Supplementary file 20 [file DataSheet15.PDF]
