# Supplementary material for: Molecular vasculogenic mimicry–Related signatures predict clinical outcomes and therapeutic responses in bladder cancer: Results from real-world cohorts
Source: Front Pharmacol. 2023 Apr 24;14:1163115. doi: 10.3389/fphar.2023.1163115 (PMC10184144; doi:10.3389/fphar.2023.1163115)

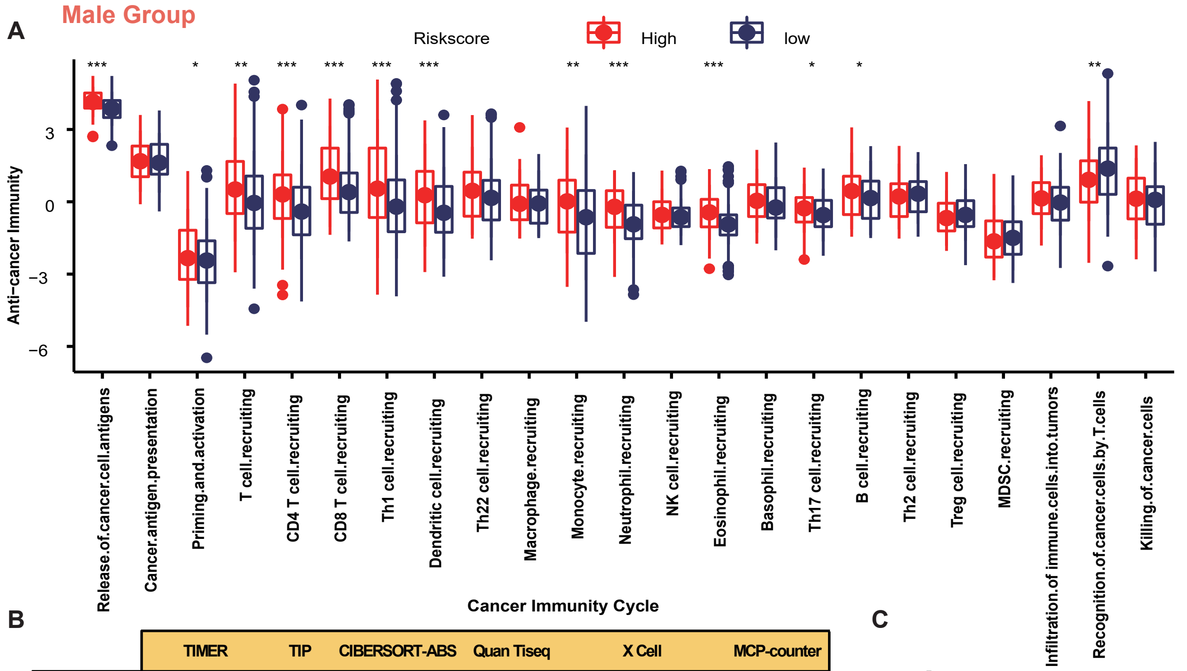

B

	TIMER	TIP	CIBERSORT-ABS	Quan Tiseq	X Cell	MCP-counter
CD8 + T_cell	0.32	Null	NS	NS	NS	NS
NK_cell	Null	NS	Activated :0.18	-0.16	NS	NS
Macrophage	0.16	Null	M0: 0.23<br>M1: 0.19<br>M2: 0.29	M1: 0.27<br>M2: 0.24	M1: 0.25<br>M2: 0.16	0.34
Dendritic cell	Myeloid DC: 0.36	NS	NS	-0.17	Activated DC: 0.25<br>Myeloid DC: 0.16<br>Plasmacytoid DC: 0.20	Myeloid DC:0.26
Th1_cell	Null	NS	Null	Null	NS	Null

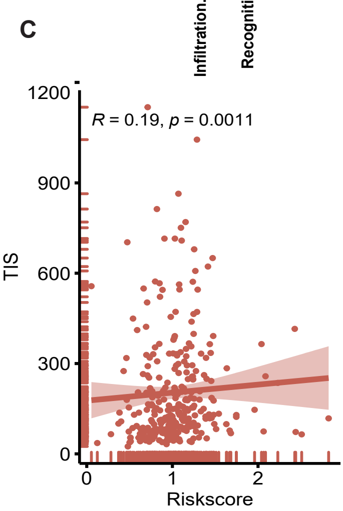

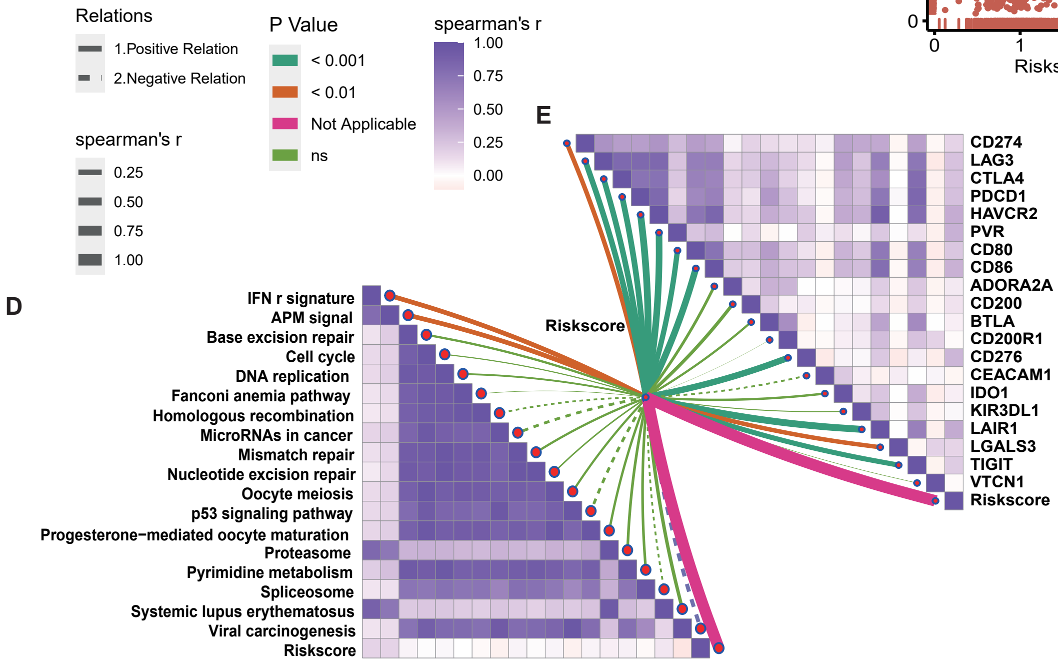

Supplement: Supplementary file 6 [file DataSheet6.PDF]
