# Supplementary material for: Molecular vasculogenic mimicry–Related signatures predict clinical outcomes and therapeutic responses in bladder cancer: Results from real-world cohorts
Source: Front Pharmacol. 2023 Apr 24;14:1163115. doi: 10.3389/fphar.2023.1163115 (PMC10184144; doi:10.3389/fphar.2023.1163115)
Supplement: Supplementary file 13 [file DataSheet1.PDF]

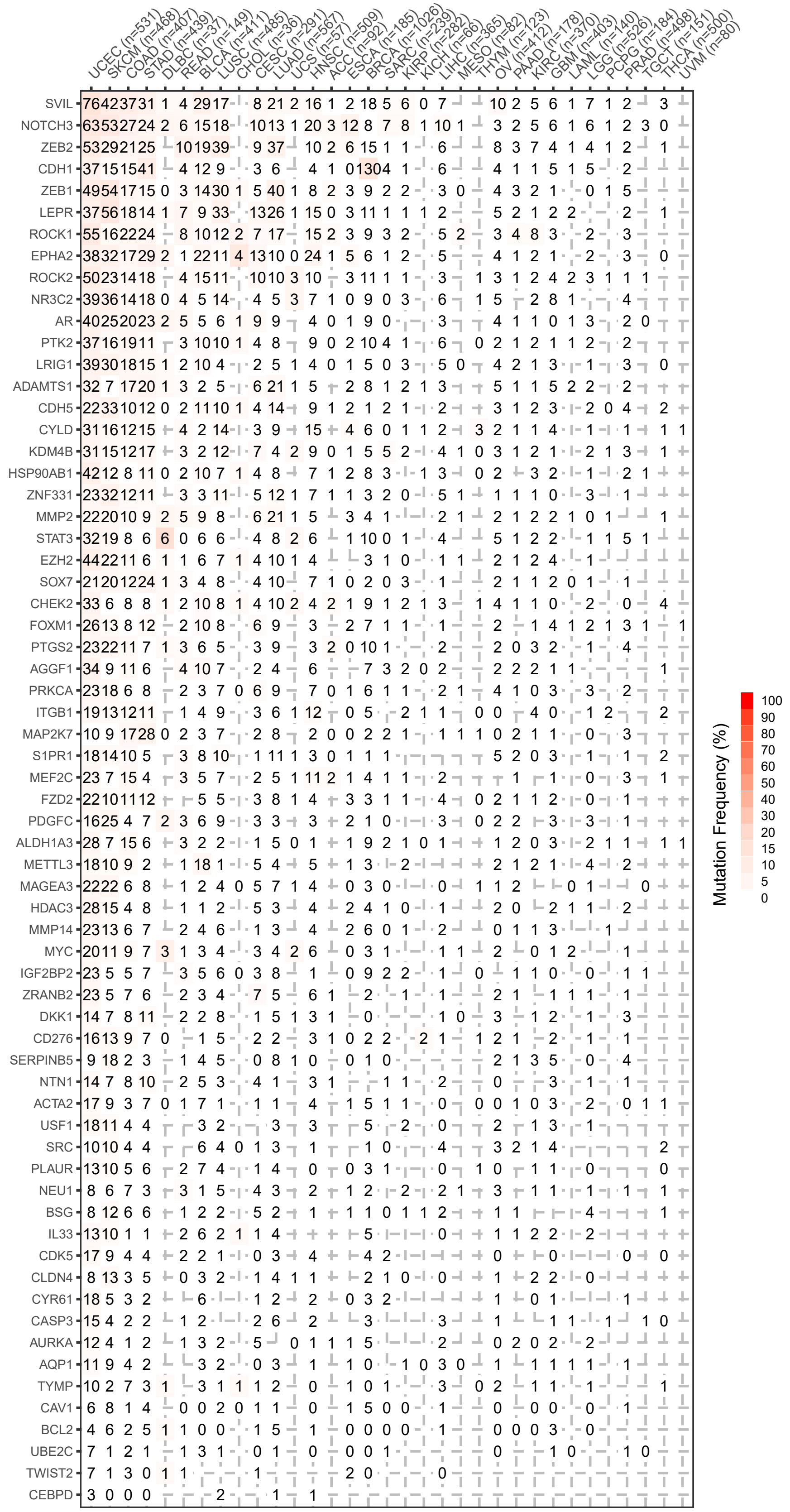
UCEC (n=531)
SKCM (n=468)
COAD (n=407)
STAD (n=439)
DLBC (n=37)
READ (n=149)
BLCA (n=411)
LUSC (n=485)
CHOL (n=36)
CESC (n=291)
LUAD (n=567)
UCS (n=57)
HNSC (n=509)
ACC (n=92)
ESCA (n=185)
BRCA (n=1026)
SARC (n=239)
KIRP (n=282)
KICH (n=66)
LIHC (n=365)
MESO (n=82)
THYM (n=123)
OV (n=412)
PAAD (n=178)
KIRC (n=370)
GBM (n=403)
LAML (n=140)
LGG (n=526)
PCPG (n=184)
PRAD (n=498)
TGCT (n=151)
THCA (n=500)
UVM (n=80)
SVIL
NOTCH3
ZEB2
CDH1
ZEB1
LEPR
ROCK1
EPHA2
ROCK2
NR3C2
AR
PTK2
LRIG1
ADAMTS1
CDH5
CYLD
KDM4B
HSP90AB1
ZNF331
MMP2
STAT3
EZH2
SOX7
CHEK2
FOXM1
PTGS2
AGGF1
PRKCA
ITGB1
MAP2K7
S1PR1
MEF2C
FZD2
PDGFC
ALDH1A3
METTL3
MAGEA3
HDAC3
MMP14
MYC
IGF2BP2
ZRANB2
DKK1
CD276
SERPINB5
NTN1
ACTA2
USF1
SRC
PLAUR
NEU1
BSG
IL33
CDK5
CLDN4
CYR61
CASP3
AURKA
AQP1
TYMP
CAV1
BCL2
UBE2C
TWIST2
CEBPD
Mutation Frequency (%)
100
90
80
70
60
50
40
30
20
15
10
5
0
