# Supplementary material for: Molecular vasculogenic mimicry–Related signatures predict clinical outcomes and therapeutic responses in bladder cancer: Results from real-world cohorts
Source: Front Pharmacol. 2023 Apr 24;14:1163115. doi: 10.3389/fphar.2023.1163115 (PMC10184144; doi:10.3389/fphar.2023.1163115)
Supplement: Supplementary file 19 [file Image1.PDF]

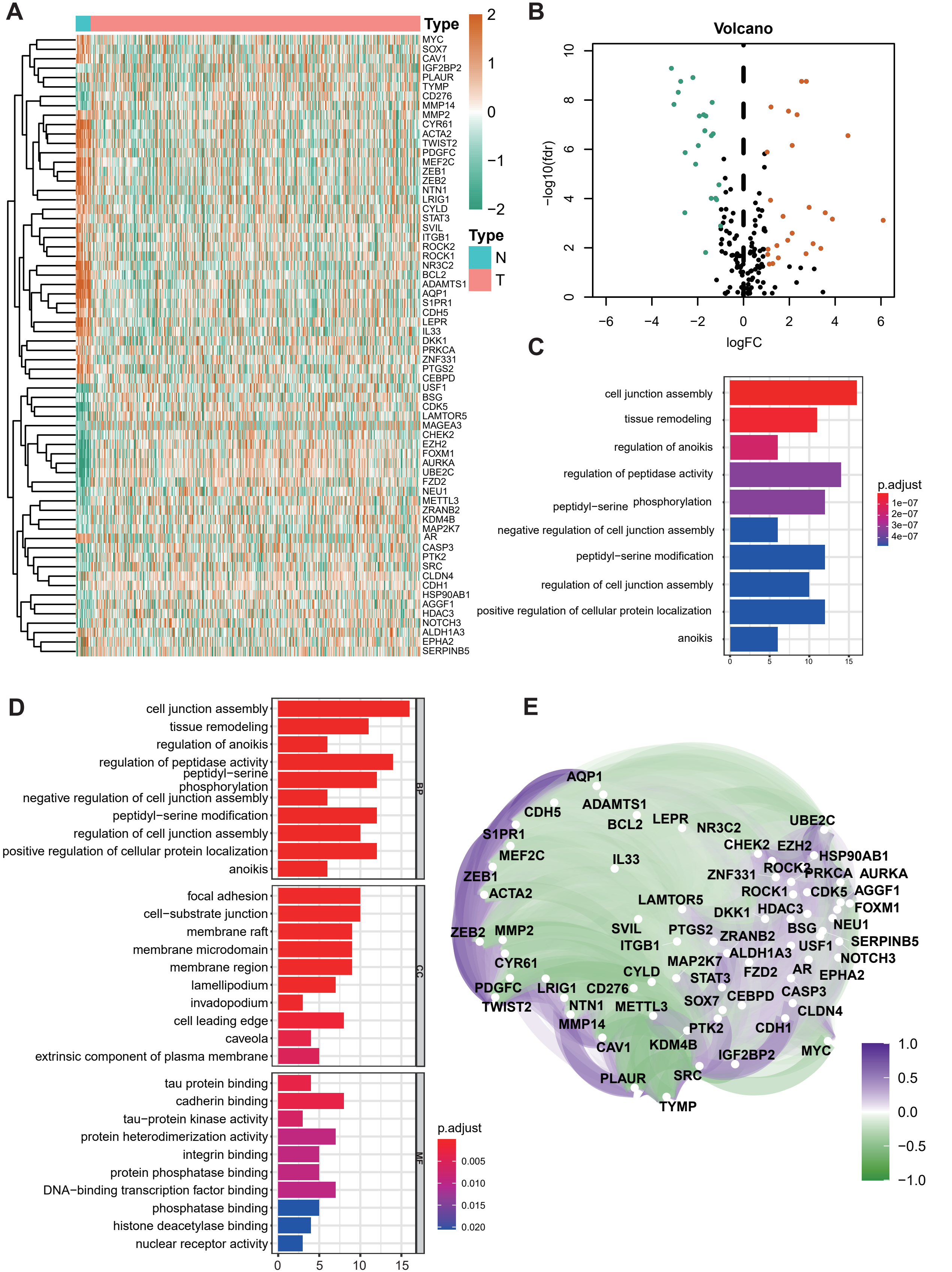
A
Type
MYC
SOX7
CAV1
IGF2BP2
PLAUR
TYMP
CD276
MMP14
MMP2
CYR61
ACTA2
TWIST2
PDGFC
MEF2C
ZEB1
ZEB2
NTN1
LRIG1
CYLD
STAT3
SVIL
ITGB1
ROCK2
ROCK1
NR3C2
BCL2
ADAMTS1
AQP1
S1PR1
CDH5
LEPR
IL33
DKK1
PRKCA
ZNF331
PTGS2
CEBPD
USF1
BSG
CDK5
LAMTOR5
MAGEA3
CHEK2
EZH2
FOXM1
AURKA
UBE2C
FZD2
NEU1
METTL3
ZRANB2
KDM4B
MAP2K7
AR
CASP3
PTK2
SRC
CLDN4
CDH1
HSP90AB1
AGGF1
HDAC3
NOTCH3
ALDH1A3
EPHA2
SERPINB5
2
1
0
−1
−2
Type
N
T
B
Volcano
−log10(fdr)
logFC
C
cell junction assembly
tissue remodeling
regulation of anoikis
regulation of peptidase activity
peptidyl−serine phosphorylation
negative regulation of cell junction assembly
peptidyl−serine modification
regulation of cell junction assembly
positive regulation of cellular protein localization
anoikis
p.adjust
1e−07
2e−07
3e−07
4e−07
D
cell junction assembly
tissue remodeling
regulation of anoikis
regulation of peptidase activity
peptidyl−serine phosphorylation
negative regulation of cell junction assembly
peptidyl−serine modification
regulation of cell junction assembly
positive regulation of cellular protein localization
anoikis
BP
focal adhesion
cell−substrate junction
membrane raft
membrane microdomain
membrane region
lamellipodium
invadopodium
cell leading edge
caveola
extrinsic component of plasma membrane
CC
tau protein binding
cadherin binding
tau−protein kinase activity
protein heterodimerization activity
integrin binding
protein phosphatase binding
DNA−binding transcription factor binding
phosphatase binding
histone deacetylase binding
nuclear receptor activity
MF
p.adjust
0.005
0.010
0.015
0.020
E
1.0
0.5
0.0
−0.5
−1.0
